# 27‐Hydroxycholesterol promotes metastasis by SULT2A1‐dependent alteration in hepatocellular carcinoma

**DOI:** 10.1111/cas.15435

**Published:** 2022-06-13

**Authors:** Taochen He, Baorui Tao, Chenhe Yi, Chong Zhang, Peng Zhang, Weiqing Shao, Yitong Li, Zhenmei Chen, Lu Lu, Huliang Jia, Wenwei Zhu, Jing Lin, Jinhong Chen

**Affiliations:** ^1^ Department of General Surgery, Huashan Hospital Fudan University Shanghai China; ^2^ Institute of Cancer Metastasis Fudan University Shanghai China

**Keywords:** 27‐hydroxycholesterol, epithelial–mesenchymal transition, HCC metastasis, oxysterol metabolism, SULT2A1

## Abstract

Oxysterol metabolism plays an important role in the initiation and development of various tumors. However, little is known that the metabolic alternation can promote the metastasis of hepatocellular carcinoma (HCC). In this study, we identify the sulfotransferase family 2A member 1 (SULT2A1) to 27‐hydroxycholesterol (27‐OHC) metabolic axis as playing a critical role in HCC metastasis. The level of 27‐OHC closely corresponded with HCC metastasis instead of proliferation in vitro and in vivo. Also, the expression of SULT2A1 is extremely downregulated in human HCC tissues and is correlated with poor prognosis and tumor metastasis. Gain‐ and loss‐of‐function studies reveal that SULT2A1 suppresses the metastasis of HCC by regulating the level of 27‐OHC. Further mechanistic studies indicated that SULT2A1‐dependent alternation of 27‐OHC activates the nuclear factor‐κB signaling pathway and promotes HCC metastasis by enhancing Twist1 expression and epithelial–mesenchymal transition. In conclusion, our findings indicate the relationship between the metabolism of 27‐OHC and the metastasis of HCC. Moreover, SULT2A1 could act as a potential prognostic biomarker and a therapeutic target for preventing HCC metastasis.

## Graphical abstract

In this study, we identify sulfotransferase family 2A member 1 (SULT2A1) to 27‐hydroxycholesterol (27‐OHC) metabolic axis as a critical role in HCC metastasis. Moreover, SULT2A1 may act as a potential prognostic biomarker and a therapeutic target for preventing HCC metastasis.

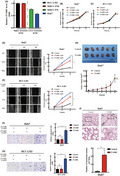

**Abbreviations**
25‐OHC, 25‐hydroxycholesterol: 27‐OHC, 27‐hydroxycholesterol; AUC, area under the receiver operating characteristic curve; DSS, disease‐specific survival; E‐cad, E‐cadherin; EMT, epithelial–mesenchymal transition; ER, estrogen receptor; GEO, Gene Expression Omnibus; HCC, hepatocellular carcinoma; LXR, liver X receptor; MVI, microvascular invasion; N‐cad, N‐cadherin; NF‐κB, nuclear factor‐κB; OS, overall survival; PVTT, portal vein tumor thrombus; ssGSEA, single sample gene set enrichment analysis; SULT, sulfotransferase; SULT2A1, sulfotransferase family 2A member 1

## 1 INTRODUCTION

Reprogramming of lipid metabolism is well known as an important characteristic of tumor cells.^1^ An accumulating amount of data has indicated the significance of cancer‐associated lipid reprogramming in tumor maintenance and establishment.^2^, ^3^ Hepatocellular carcinoma is a main leading cause of cancer‐related death worldwide.^4^, ^5^ Much evidence suggests that the dysfunction of lipid and cholesterol metabolisms is involved in the progression and metastasis of HCC.^6^, ^7^ Therefore, understanding the relationship between the metabolism and HCC is very important.

Oxysterols are oxidized forms of cholesterol or of its precursors. Constituting a large family of lipids (i.e., the oxysterome), they are mainly produced through two pathways, enzymatic or nonenzymatic oxidation reaction.^8^ Previously, it has been shown that dysfunction of oxysterol can lead to metabolic, inflammatory, and neurodegenerative diseases,^9^, ^10^, ^11^ involving many physiological processes such as membrane fluidity, membrane protein activity,^12^ vesicle trafficking, and cytoskeleton function.^12^, ^13^ 27‐Hydroxycholesterol, an important member of the oxysterols family, has received less attention but is increasingly being recognized in cancer cells. It has been reported that 27‐OHC can show pro‐oncogenic effects through regulating inflammation and several signaling pathways,^14^ or through oxysterol‐binding proteins.^15^ 27‐Hydroxycholesterol can mainly be metabolized by sulfation. Sulfotransferases are considered to be exclusive enzymes for sulfidic oxysterols.^16^, ^17^ However, their roles in oxysterol metabolism as well as in cancer development remains uncertain.

Metastasis is a significant hallmark of cancer and leads to most HCC‐related deaths.^18^ To date, several researchers have verified the relationship between specific metabolic alternations and HCC metastasis.^19^, ^20^ In this study, we identify the SULT2A1–27‐OHC metabolic axis as a key player in HCC metastasis instead of proliferation. Moreover, considered as a prognostic biomarker and a therapeutic target, its potential value might reduce the mortality caused by HCC metastasis.

## 2 MATERIALS AND METHODS

Detailed Material and Methods are shown in Appendix S1.

## 3 RESULTS

### 3.1 27‐Hydroxycholesterol can increase HCC metastasis without affecting cell proliferation

**FIGURE 1:**
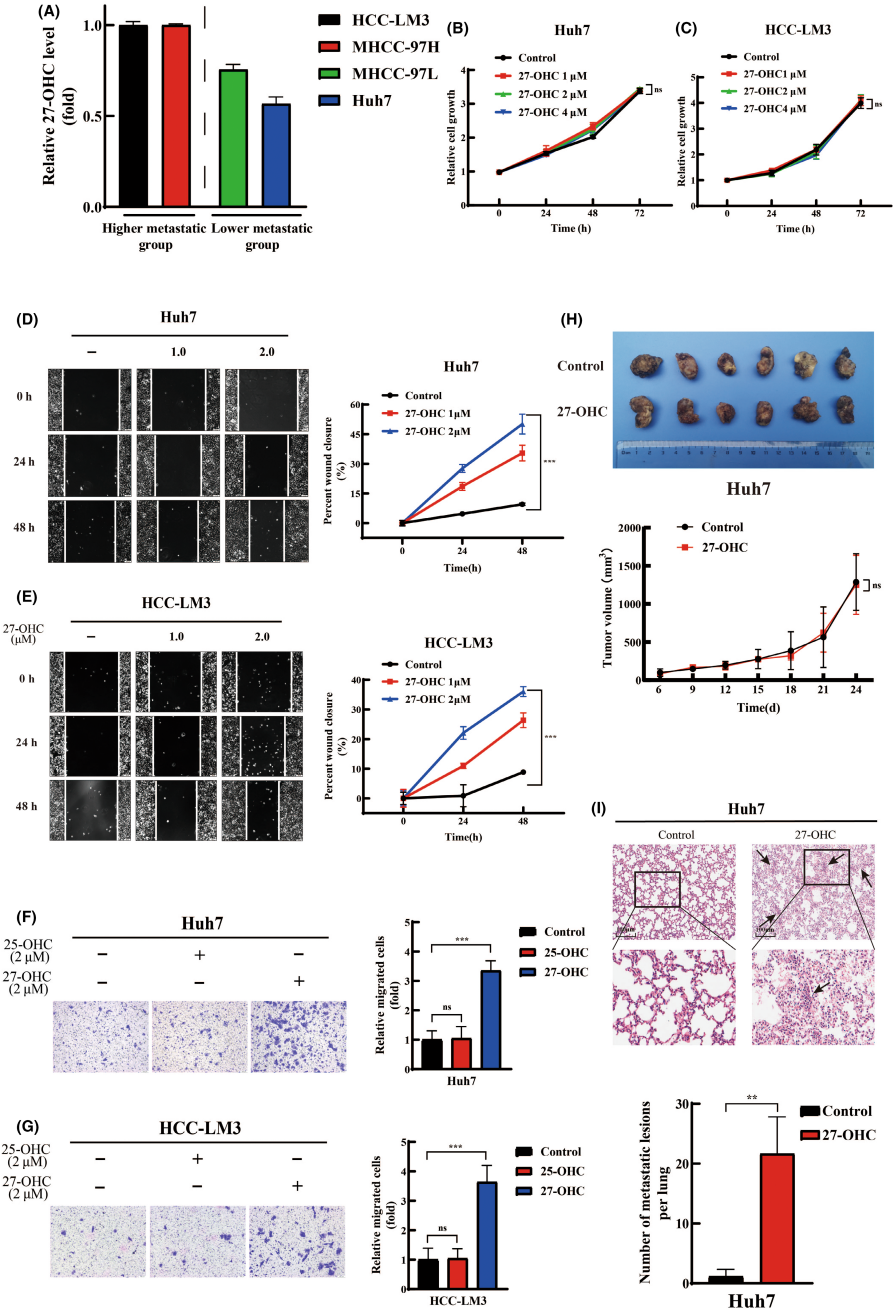
27‐Hydroxycholesterol (27‐OHC) increases hepatocellular carcinoma (HCC) metastasis in vitro and in vivo. (A) Levels of 27‐OHC in four common HCC cell lines which were divided into two groups based on their metastatic potential. Levels were determined by ELISA. (B–E) Effects of 27‐OHC on in vitro proliferation (B, C, CCK‐8 assays) and migration (D, E, wound scratch assay) of HCC cells. Significance was determined by two‐way ANOVA (Bonferroni post test). Scale bar, 100 μm. (F, G) Effects of 25‐OHC and 27‐OHC on in vitro invasion of HCC cells (Transwell assay). Significance was determined by Student’s *t*‐test. Scale bar, 100 μm. (H, I) Effects of 27‐OHC in vivo on tumor volume in subcutaneous xenograft models (H) or the spontaneous lung metastasis in orthotopic xenograft models (I). In (I), arrows refer to lung metastatic lesions. Scale bar, 100 μm. Significance was determined by two‐way ANOVA (Bonferroni post test) (H, bottom panel) or Wilcoxon test (I, bottom panel). *p < 0.05, **p < 0.01, ***p < 0.001. ns, not significant

First, we evaluated the association between 27‐OHC and HCC in vitro. To make our results more reliable, we chose 25‐OHC, a similar member of the oxysterol family, as a control. We analyzed the intracellular oxysterols in four common human HCC cell lines (MHCC‐97H, HCC‐LM3, MHCC‐97L, and Huh7) with different metastatic potential^21^ by ELISA and found that the levels of 27‐OHC and 25‐OHC were obviously increased in the higher metastasis group compared the lower metastasis group (Figures 1A and S1A). To further validate the relationship between oxysterols and HCC, we evaluated their functions in in vitro cell proliferation, migration, and invasion of Huh7 and HCC‐LM3 cells with the addition of different concentrations of 27‐OHC or 25‐OHC. The levels of 27‐OHC along with 25‐OHC did not show positive effects on tumor proliferation (Figures 1B,C and S1B,C). However, 27‐OHC significantly enhanced the migration ability of Huh7 and HCC‐LM3 cells, which was strictly dependent on concentration, whereas 25‐OHC showed no similar effects (Figures 1D,E and S1D,E). Similar phenomena were observed when considering the invasion ability (Figure 1F,G).

To verify these findings, the effects of 27‐OHC were further evaluated on in vivo tumor growth and metastasis of HCC xenografts. 27‐Hydroxycholesterol did not show an obvious effect on tumor growth of HCC xenografts in subcutaneous xenograft models (Figure 1H). In orthotopic xenograft models, the corresponding HCC subcutaneous xenografts were isolated and implanted into the liver. The 27‐OHC group was also treated with 27‐OHC 20 mg/kg i.p. every 3 days. Mice was killed and the primary tumors and lungs were resected after 6 weeks (Figure S1F). Serial sections of every lung tissue were taken and stained with H&E to determine lung metastasis. Almost no lung metastasis lesions were found in nude mice bearing orthotopic xenografts from the control group. However, we observed a significant increase in lung metastasis in nude mice bearing orthotopic xenografts from the 27‐OHC treated group (Figure 1I). These data suggest a close relationship between 27‐OHC and metastasis of HCC.

### 3.2 Low expression of SULT2A1 is associated with poor prognosis of HCC patients

**FIGURE 2:**
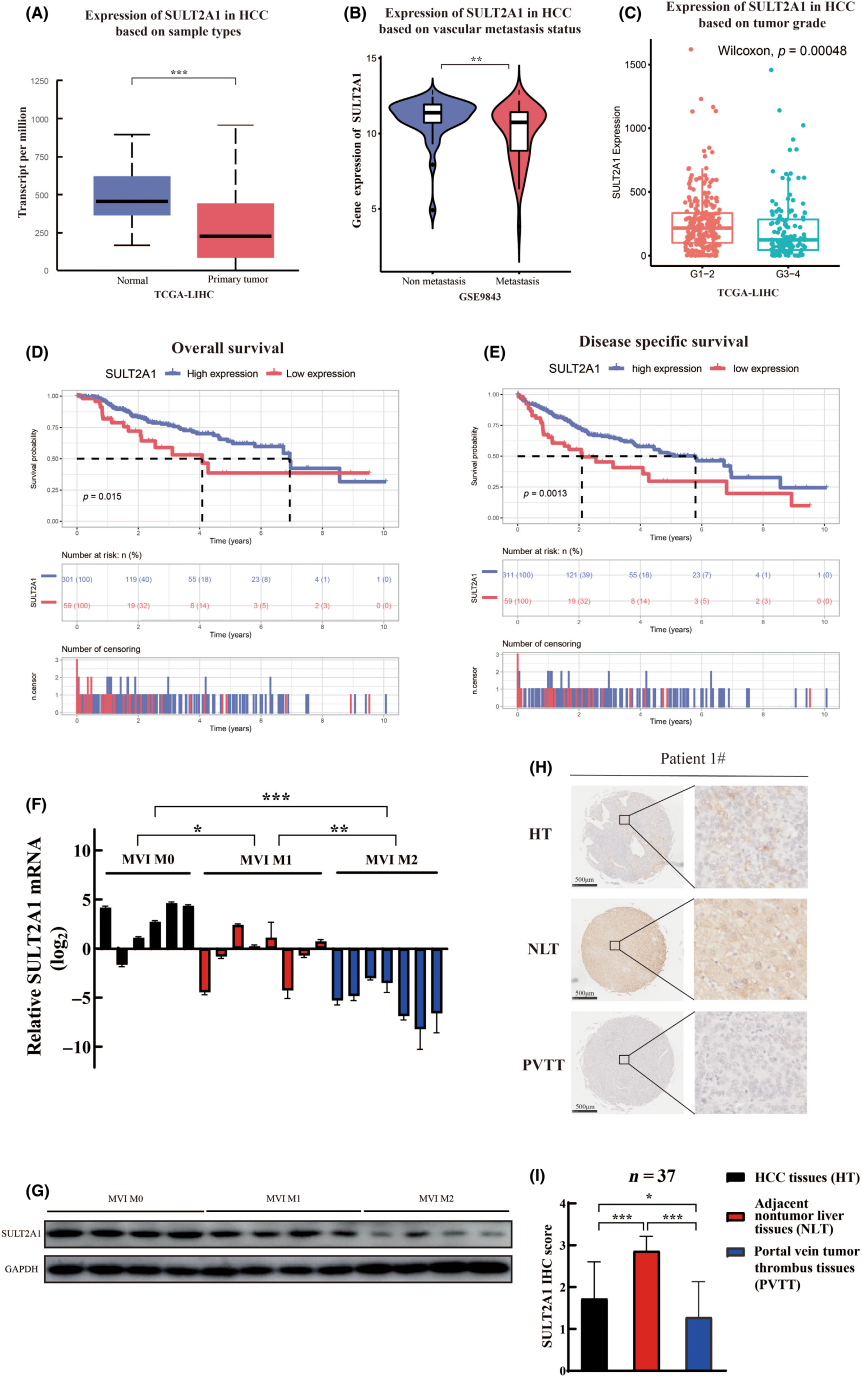
Low expression of sulfotransferase family 2A member 1 (SULT2A1) is correlated with hepatocellular carcinoma (HCC) metastasis and clinical characteristics. (A) Expression of SULT2A1 in HCC tissues compared with normal tissues based on The Cancer Genome Atlas Liver Hepatocellular Carcinoma (TCGA‐LIHC) database. All data were obtained from TCGA (https://portal.gdc.cancer.gov/). (B) Expression of SULT2A1 in HCC vascular invasion (metastasis) compared with nonvascular invasion (nonmetastasis) tissues based on the Gene Expression Omnibus (GEO) dataset (GSE9843). All data were obtained from the GEO database (https://www.ncbi.nlm.nih.gov/geo/ Home ‐ GEO ‐ NCBI (nih.gov)). (C) Expression of SULT2A1 in grade 1–2 HCC compared with grade 3–4 HCC based on the TCGA‐LIHC database. (D, E) Prognostic significance of SULT2A1 for HCC patients from the TCGA database assessed by Kaplan–Meier analysis. Patients with low SULT2A1 expression have poorer overall survival (D) and poorer disease‐specific survival (E) than patients with high SULT2A1 expression. (F) Quantitative real‐time PCR analysis of SULT2A1 mRNA levels in human HCC tissues grouped by microvascular invasion (MVI) grade (MVI M0, *n* = 6; M1, *n* = 8; M2, *n* = 7). Data are shown after log transformation. Significance was determined using Student’s *t*‐test. (G) Immunoblot of SULT2A1 protein in human HCC tissues of different MVI grades (*n* = 4 for each group). (H, I) Levels of SULT2A1 from paired HCC tissues, adjacent nontumor liver tissues (NLT), and portal vein tumor thrombus tissues (PVTT) based on tissue microarray. Representative images of immunohistochemical (IHC) staining from one patient (H) and the IHC H scores (I) are shown. *n* = 37. Significance was determined using Student’s *t*‐test. *p < 0.05, **p < 0.01, ***p < 0.001. ns, not significant

Next, we examined the possible influencing factor of 27‐OHC in HCC. As previously mentioned, SULT, mainly SULT2A1, SULT2B1, and SULT1E1, are responsible for oxysterol metabolism.^16^, ^22^ We analyzed the former three enzymes at the transcriptional level using public databases. Data revealed that, compared to the other two enzymes, SULT2A1 showed significant decrease in tumor tissues against normal control (Figures 2A and S2A,B). Furthermore, the expression of SULT2A1 was similarly reduced in HCC vascular metastasis tissues compared with nonvascular metastasis tissues (Figures 2B and S2C,D), which might suggest the inherent association with HCC. We also analyzed the expression of SULT2A1 among different tumor grades and found that downregulation of SULT2A1 was correlated with highly malignant tumor, consistent with previous findings (Figure 2C).

We then undertook survival analyses to determine whether downregulation of SULT2A1 might affect prognosis. Using Kaplan–Meier survival analysis, low expression of SULT2A1 was shown to be significantly correlated with worse OS (*p* = 0.015) and DSS (*p* = 0.0013) (Figure 2D,E).

We also used another public cohort to further verify our previous findings. Based on the GEO database (GSE14520), HCC patients with lower expression of SULT2A1 were considered to have worse tumor types and poorer clinical outcomes, which were similar to the above results (Figure S2E–G).

Subsequently, the clinical association of SULT2A1 expression was examined in our own patients’ set by using quantitative real‐time PCR and western blotting. It was revealed that both mRNA and protein levels of SULT2A1 were much lower in HCC tissues compared with the paired adjacent nontumor liver tissues (Figure S3A,B). Furthermore, the cases were grouped according to the MVI grade. Data showed that low expression of SULT2A1 corresponded to a high probability of MVI occurrence (Figure 2F,G). We also examined the expression of SULT2A1 in the previous tissue microarray^23^ containing paired primary HCC tissues, adjacent nontumor liver tissues and PVTT tissues from our research group. Similarly, results showed that PVTT tissues, representing HCC metastasis, had lower expression of SULT2A1 compared with HCC and nontumor liver tissues (Figure 2H,I).

We then measured the expression of SULT2A1 among HCC cell lines and found that the mRNA and protein levels of SULT2A1 were significantly associated with their metastatic potential (Figure S3C,D). Consequently, these results strongly suggest that downregulation of SULT2A1 is obviously correlated with poor prognosis of HCC patients and further indicate that SULT2A1 might play a vital role in the metastasis of HCC.

### 3.3 Downregulation of SULT2A1 can increase the metastasis of HCC by modulating the level of 27‐OHC

**FIGURE 3:**
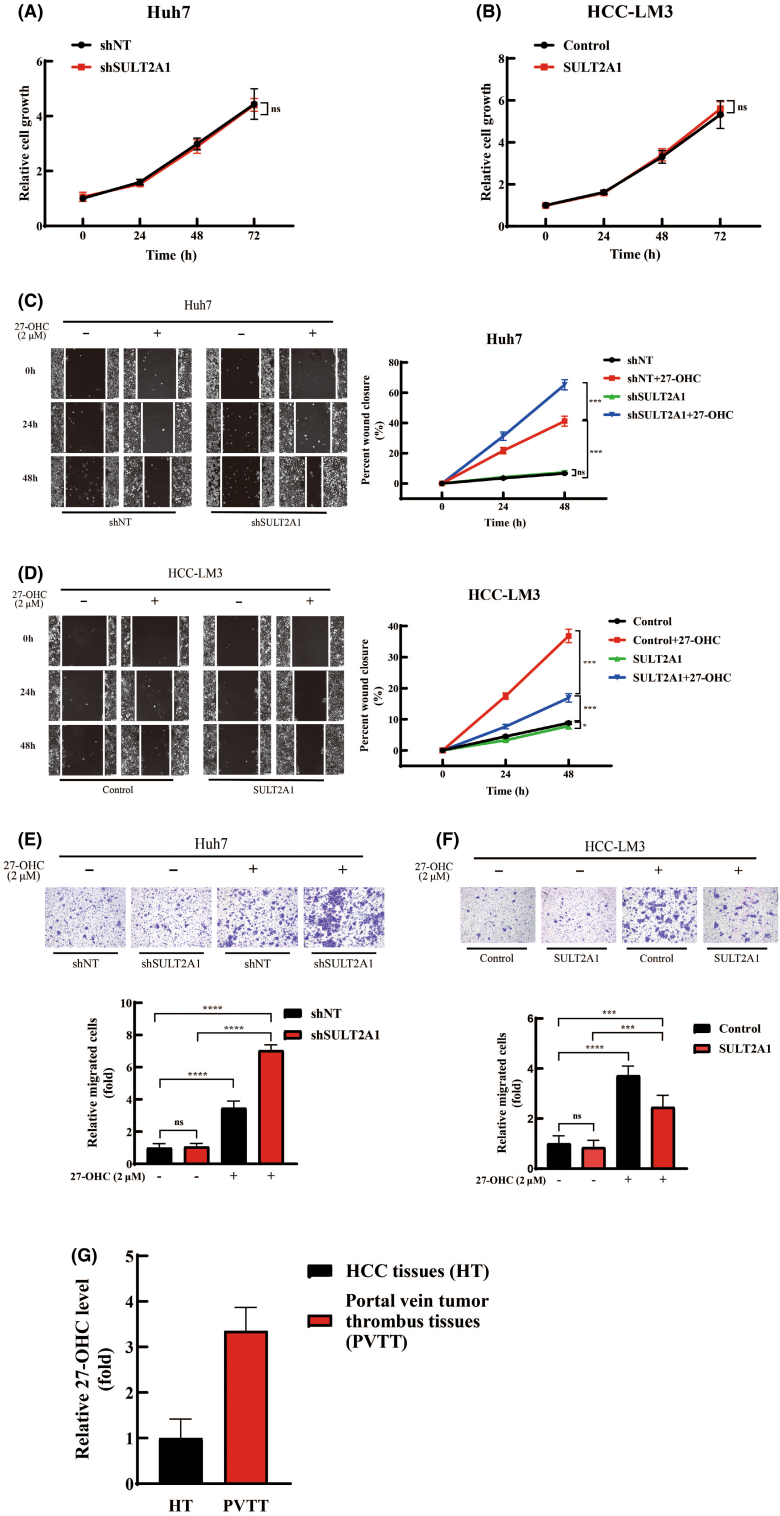
Sulfotransferase family 2A member 1 (SULT2A1)‐dependent alternation of 27‐hydroxycholesterol (27‐OHC) is vital for hepatocellular carcinoma (HCC) metastasis in vitro. (A, B) Effects of SULT2A1 gain‐ and loss‐of‐function on in vitro proliferation (CCK‐8 assay) of HCC cells. Relative cell number was expressed as fold change to Time 0 h. (C–F) Effects of SULT2A1 gain‐ and loss‐of‐function on in vitro migration (C, D, wound scratch assay) and invasion (E, F, Transwell assay) of HCC cells. shSULT2A1 and SULT2A1 groups were constructed as described previously and were treated with or without 2 μM 27‐OHC for 72 h. Scale bar, 100 μm. (G) Levels of 27‐OHC in primary human HCC tissues (HT) and human portal vein tumor thrombus tissues (PVTT). Levels were determined by ELISA. Significance was determined by two‐way ANOVA (Bonferroni post test) (A, B, and right panels of C, D) or Student’s *t*‐test (E–G, bottom panels). *p < 0.05, **p < 0.01, ***p < 0.001. ns, not significant

We undertook in vitro functional studies to determine the roles of SULT2A1 and 27‐OHC in HCC cells. Two SULT2A1‐specific shRNAs were generated to silence SULT2A1 expression (shSULT2A1). shSULT2A1#1, which induced a more significant knockdown effect, was adopted for knocking down the SULT2A1 expression in Huh7 cells that highly expressed SULT2A1 in previous studies (shown in Figures S3 and S4A,B). We also overexpressed SULT2A1 in HCC‐LM3 cells that expressed SULT2A1 at low levels. First, we verified the relationship between SULT2A1 and 27‐OHC in the HCC‐LM3 and Huh7 modulated cells. The level of intracellular 27‐OHC displayed an opposite change with the expression of SULT2A1, demonstrating the metabolism effect of the enzyme to the substrate (Figure S4C,D). Overexpression or knockdown of SULT2A1 did not show a positive impact on the proliferation of HCC (Figure 3A,B). In the wound scratch assay, we failed to observe an obvious difference between the control group and the modulated group initially due to the lack of 27‐OHC in the ordinary in vitro culture environment with 10% FBS DMEM. However, differences became apparent when exogenous 27‐OHC was added. Silencing of SULT2A1 strongly stimulated the migration capabilities of Huh7 cells, whereas overexpression of SULT2A1 resulted in a significant inhibition of migration of HCC‐LM3 cells (Figure 3C,D). Similar results were also obtained in the Transwell assay (Figure 3E,F). We further measured the level of 27‐OHC in human tumors and found it higher in PVTT tissues compared with primary HCC tissues (Figure 3G), These studies again prove the vital status of 27‐OHC in HCC invasion and migration and indicate that the existence of 27‐OHC is required for the prometastasis effect of downregulating SULT2A1.

### 3.4 Downregulation of SULT2A1 and increase of 27‐OHC lead to EMT in HCC

**FIGURE 4:**
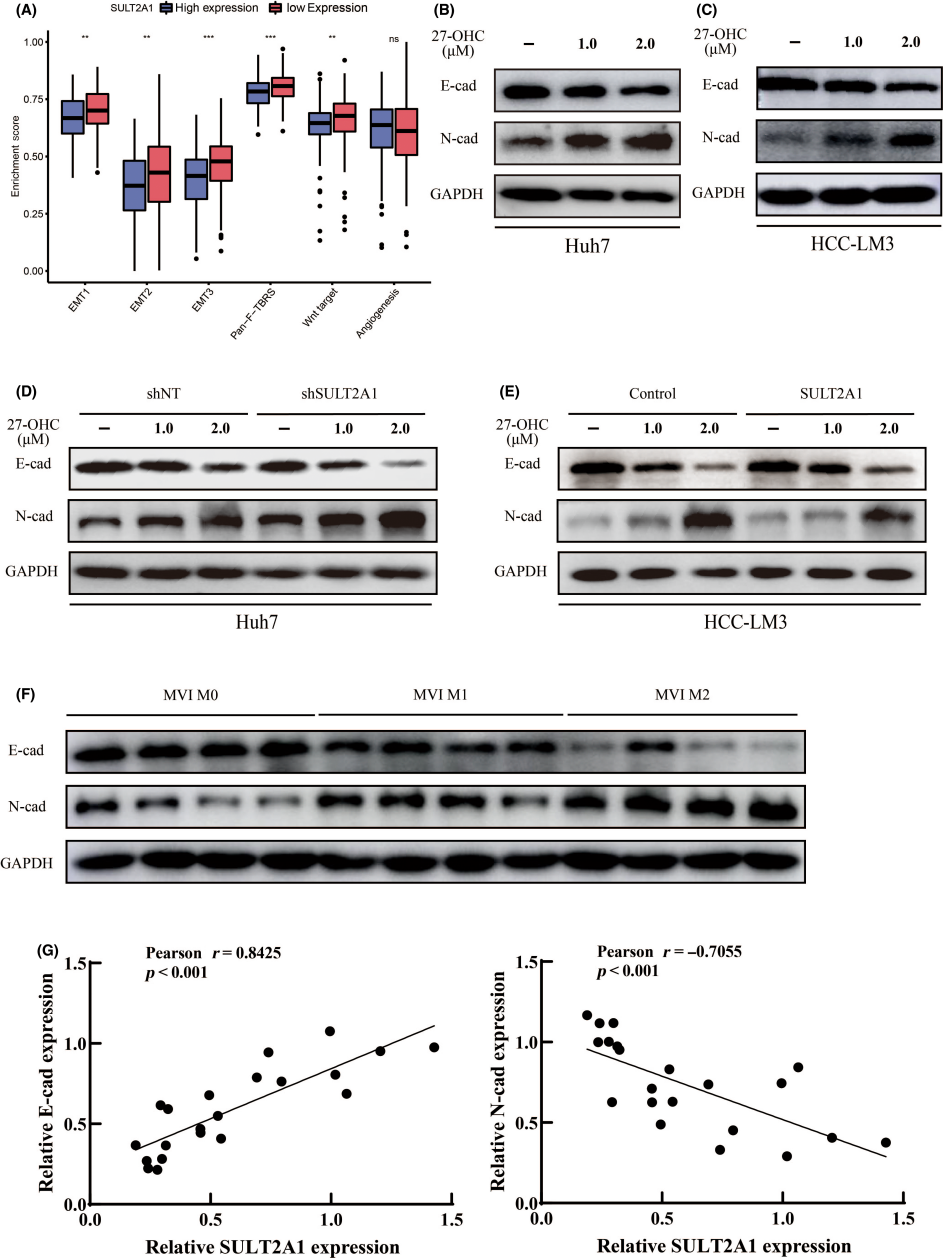
Sulfotransferase family 2A member 1 (SULT2A1)‐dependent alternation of 27‐hydroxycholesterol (27‐OHC) leads to epithelial–mesenchymal transition (EMT) in hepatocellular carcinoma HCC. (A) Enrichment scores of stromal‐activation relevant signatures between different expression groups of SULT2A1 based on single sample gene set enrichment analysis (ssGSEA). The median SULT2A1 expression was used as a cut‐off value. All data were obtained from The Cancer Genome Atlas (TCGA) dataset. Details are shown in Tables S1 and S2. (B, C) Expression of EMT markers in Huh7 (B) and HCC‐LM3 (C) cells treated with different concentrations of 27‐OHC for 72 h was determined by western blotting analysis. (D, E) Expression of EMT markers in SULT2A1‐shRNA stably expressed Huh7 (D) or in SULT2A1 stably expressed HCC‐LM3 (E) cells treated with different concentrations of 27‐OHC for 72 h was determined by western blotting analysis. (F) Expression of EMT markers in human HCC tissues with different grades of microvascular invasion and SULT2A1 expression was determined by western blot analysis. (G) Correlation between expression of SULT2A1 and EMT markers in HCC tissues based on the result of western blot analyses. Gray scale values of bands were analyzed by ImageJ software. The correlation was then analyzed by Pearson’s correlation analysis. ***p < 0.001. For (B–F), each experiment was carried out in triplicate. Representative results are shown. E‐cad, E‐cadherin; N‐cad, N‐cadherin

Epithelial–mesenchymal transition is well known as a promoting mechanism in HCC metastasis.^24^, ^25^ We next evaluated whether SULT2A1‐dependent alteration of 27‐OHC could affect EMT. We collected several stromal activation‐relevant signatures from published reports and analyzed their enrichment scores by ssGSEA based on different databases. Data revealed that low expression of SULT2A1 is significantly correlated with EMT‐activated relevant signatures (Figures 4A and S5A). We then examined the expression of two key proteins, E‐cad and N‐cad, in HCC cells by western blotting and found that HCC could acquire the capacity to migrate with the addition of 27‐OHC. Furthermore, the gain of ability was dependent on the 27‐OHC concentration (Figure 4B,C). For control, we identified the state of EMT in HCC cells treated with 25‐OHC and no difference was found among different concentrations compared with those treated with 27‐OHC (Figure S5B,C). Interestingly, we also found an obvious change in Huh7 cells with 27‐OHC treatment (Figure S5D). Huh7 cells seemed to homogeneously lose tight intercellular junctions, and changed into smaller, partly long spindle‐shaped ones. This evidence provides strong proof for the activation of EMT in HCC with 27‐OHC.

To verify whether the modulation of SULT2A1 had influence in 27‐OHC affecting EMT, we further examined the change of EMT‐related protein in Huh7 and HCC‐LM3 modulated cells. After knockdown of SULT2A1, the expression of E‐cad was obviously decreased, whereas the expression of N‐cad was increased in Huh7 cells (Figure 4D). When SULT2A1 was upregulated in HCC‐LM3 cells, the expression of E‐cad was increased, and the expression of N‐cad was significantly reduced (Figure 4E). All the results could only be proven given the existence of the equal concentration of 27‐OHC.

We also checked the EMT status in human HCC tissues with different SULT2A1 expressions. Similar results were found, in that human HCC with lower SULT2A1 expressions (MVI M1‐2, based on Figure 2G) had higher EMT levels (Figure 4F). More importantly, the expression of SULT2A1 and EMT markers was highly correlated by using gray analysis (SULT2A1 and E‐cad: Pearson’s *r* = 0.8425; SULT2A1 and N‐cad: Pearson’s *r* = −0.7055, *p* < 0.001) (Figure 4G). Collectively, these data show that SULT2A1‐dependent alternation of 27‐OHC promotes the migration of HCC by regulating EMT.

### 3.5 Sulfotransferase 2A1‐dependent alternation of 27‐OHC is correlated with activation of NF‐κβ signaling pathway, together with elevated Twist1 expression in HCC

**FIGURE 5:**
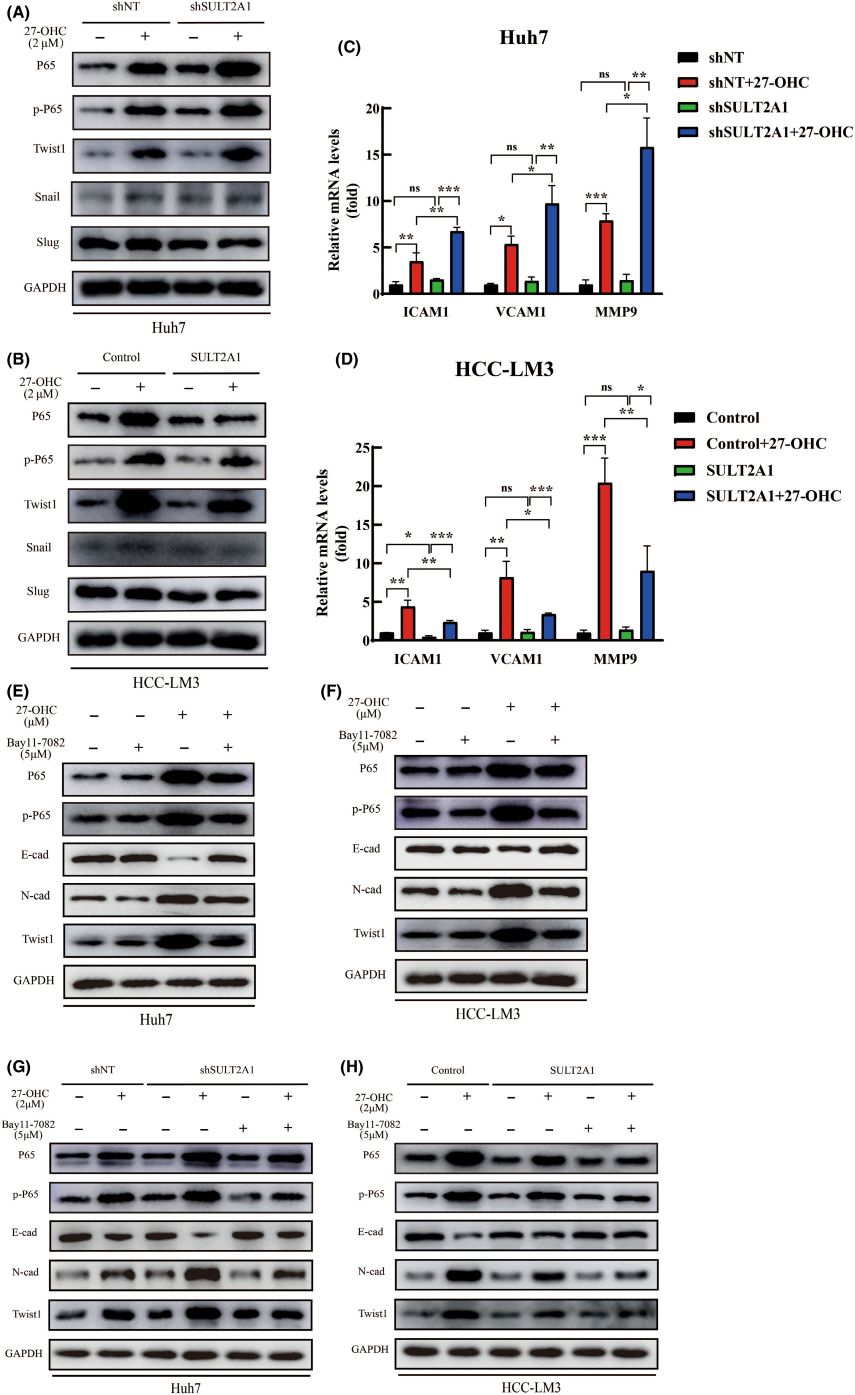
Sulfotransferase family 2A member 1 (SULT2A1)‐dependent alternation of 27‐hydroxycholesterol (27‐OHC) is correlated with the activation of the nuclear factor‐κB (NF‐κβ) signaling pathway, together with elevated Twist1 expression in hepatocellular carcinoma (HCC). (A, B) Expression of p65, p‐p65, and epithelial–mesenchymal transition (EMT)‐related transcription factors were determined by western blotting analysis. Huh7 (including shSULT2A1 group) (A) and HCC‐LM3 (including SULT2A1 group) (B) were treated with or without 2 μM 27‐OHC for 72 h. (C, D) Several downstream genes of the NF‐κB signaling pathway which connected with tumor migration were evaluated by Quantitative RT‐PCR analysis after treatment with 27‐OHC in SULT2A1‐shRNA stably expressed Huh7 (C) and SULT2A1 overexpressed HCC‐LM3 (D) cells. *p < 0.05, **p < 0.01, ***p < 0.001. ns, not significant. (E, F) Expression of p65, p‐p65, Twist1, and EMT markers in Huh7 (E) and HCC‐LM3 (F) cells were determined by western blot analysis. All groups were treated with or without 2 μM 27‐OHC and 5 μM Bay11‐7082 for 72 h. (G, H) Expression of p65, p‐p65, Twist1, and EMT markers were determined by western blot analysis. Huh7 (including shSULT2A1 group) (G) and HCC‐LM3 (including SULT2A1 group) (H) were treated with or without 2 μM 27‐OHC and 5 μM Bay11‐7082 for 72 h

As inflammation is critically involved in the pathological progress between oxysterols and diseases, we evaluated the impact of 27‐OHC on inflammation. Nuclear factor‐κB signaling pathway is widely acknowledged as an essential role in inflammation. In recent years, it has also been increasingly realized as a crucial player in many steps of cancer initiation and progression.^26^, ^27^ Moreover, much research has been focused on the relationship between NF‐κB and EMT. We next evaluated whether 27‐OHC could affect EMT in HCC cells through the NF‐κB signaling pathway. Two key factors, NF‐κB p65 and phospho‐NF‐κB p65 (Ser536) (p‐p65), were examined by western blotting with or without the addition of 27‐OHC in two HCC cell lines. Data showed that when treated with 27‐OHC, the expression of p65 and p‐p65 were both increased. In Huh7 and HCC‐LM3 modulated cells, the reduced SULT2A1 expression obviously increased the expression of p65 and p‐p65, and the upregulation of SULT2A1 reversed this alternation following 27‐OHC treatment (Figure 5A,B). We further evaluated several downstream genes of the NF‐κB signaling pathway^28^, ^29^ that are connected with tumor migration, and found similar changes corresponding with p‐65 and p‐p65 as described in Figure 5A,B (Figure 5C,D). These results indicate that SULT2A1 affects the NF‐κB signaling pathway by altering 27‐OHC level.

Twist1, Snail, and Slug, transcription factors associated with EMT, were also examined in cell lines treated with 27‐OHC or not (Figure 5A,B). Compared with others, the expression of the transcription factor Twist1 were significantly changed along with p65 in both Huh7 and HCC‐LM3 cell lines, indicating the intrinsic connection between NF‐κB and EMT.

To further explore our findings between 27‐OHC, NF‐κB, and EMT, a small molecular inhibitor of NF‐κB, Bay11‐7082, was used in the following studies.^30^ Bay11‐7082 did not show significant influence on HCC migration in WT Huh7 or HCC‐LM3 cells without 27‐OHC. However, the changes of E‐cad and N‐cad expression resulting from treatment with 27‐OHC were reversed through the inhibition of the NF‐κB signaling pathway. Similar results were also observed in consideration of Twist1 (Figure 5E,F). These data indicate that 27‐OHC affects EMT in HCC by regulating the NF‐κB signaling pathway and elevating the level of Twist1.

We then verified our findings in the SULT2A1‐modulated cells of Huh7 and HCC‐LM3 cell lines. In Huh7 cells, the alternation of p65 and p‐p65 caused by the downregulation of SULT2A1 under 27‐OHC treated circumstance were reversed by the addition of Bay11‐7082, together with EMT‐related transcription factors and proteins. In HCC‐LM3 cells treated with 27‐OHC, the inhibitor further strengthened the changes of NF‐κB and EMT suppressed by the upregulation of SULT2A1 (Figure 5G,H). Consequently, these results strongly support a positive relationship between the downregulation of SULT2A1, the level of 27‐OHC, activation of NF‐κβ, and Twist1 expression in human HCCs.

### 3.6 Upregulation of SULT2A1 inhibits HCC metastasis by suppressing NF‐κB signaling pathway and EMT phenotype in vivo

**FIGURE 6:**
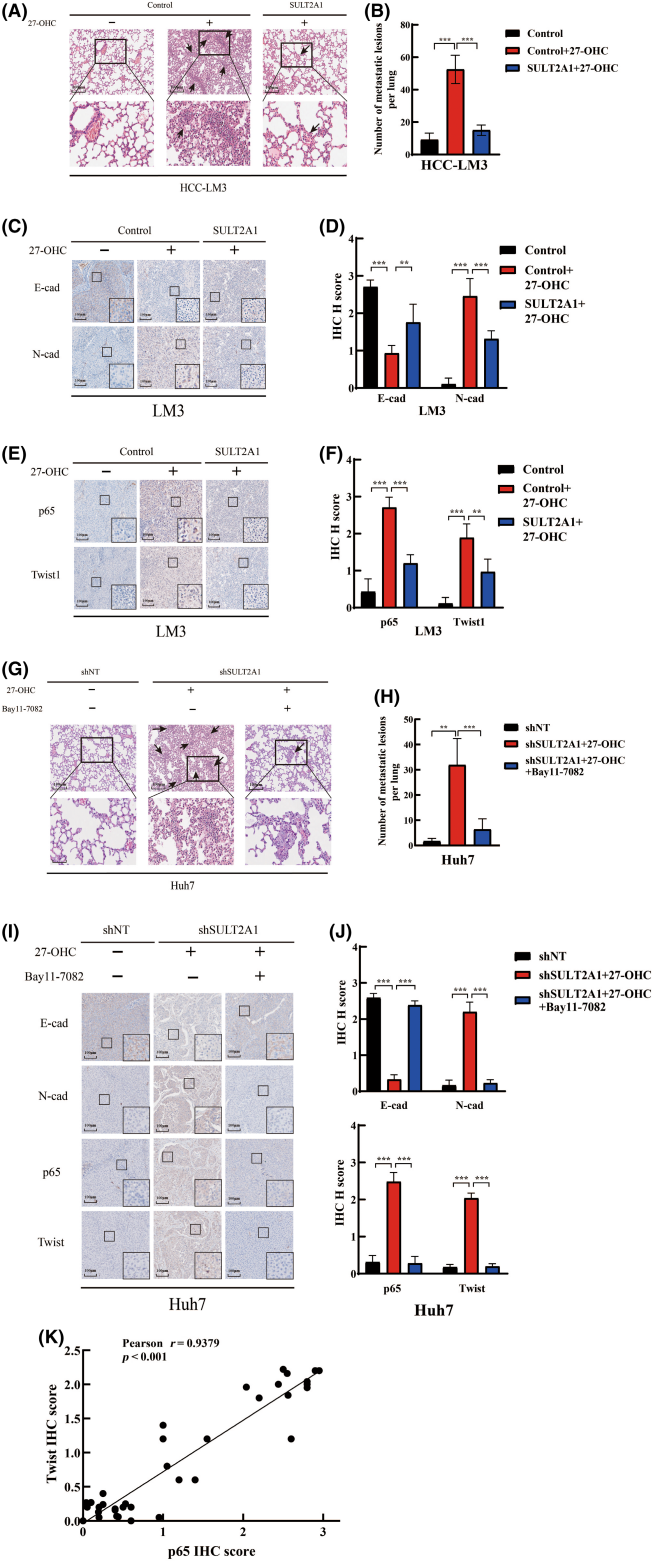
Upregulation of sulfotransferase family 2A member 1 (SULT2A1) suppresses the nuclear factor‐κB (NF‐κB) signaling pathway and epithelial–mesenchymal transition (EMT) phenotype in vivo, leading to the inhibition of hepatocellular carcinoma (HCC) metastasis. (A, B) SULT2A1‐overexpressed HCC‐LM3 cells and the control group treated with 27‐OHC or not were used to establish orthotopic xenograft models. Representative H&E staining images of lung tissues (A) and the average numbers of lung metastatic lesions (B) from six mice per group are shown. Arrows refer to lung metastatic lesions. (C–F) Levels of EMT markers (C, D) and p65 and Twist1 (E, F) from SULT2A1‐overexpressed HCC tissues and the control group were analyzed by immunohistochemistry (IHC). Representative images of IHC staining from six mice per group and the IHC H scores are shown. (G, H) shSULT2A1 Huh7 cells and the shNT group treated with 27‐OHC or Bay11‐7082 were used to establish orthotopic xenograft models. Representative H&E staining images of lung tissues (G) and the average numbers of lung metastatic lesions (H) from six mice per group are shown. Arrows refer to lung metastatic lesions. (I, J) Levels of EMT markers and p65 and Twist1 from shSULT2A1 HCC tissues and the shNT group were analyzed by IHC. Representative images of IHC staining from six mice per group (I) and the IHC H scores (J) are shown. Scale bar, 100 μm (A, C, E, G, I). Significance was determined by Student’s *t*‐test (B, D, F, H, J). *p < 0.05, **p < 0.01, ***p < 0.001. ns, not significant. K, Correlation between p65 expressions and Twist1 expression in HCC tissues based on the IHC H score. The correlation was then analyzed by Pearson correlation analysis. ***p < 0.001. shNT, shRNA non‐target

A lung metastasis mouse model was adopted to verify our previous in vitro findings of metastasis of HCC, similar to that shown in Figure 1I. Differently, both SULT2A1 overexpressing cells and the control WT cells derived from the HCC‐LM3 cell line were used in the construction of the model. Upregulation of SULT2A1 reversed the ability of metastasis contributed by the presence of 27‐OHC (Figure 6A,B). Immunohistochemical staining of mouse tumor tissues in livers was also carried out to detect the relationship between 27‐OHC, SULT2A1, and EMT. The expression of E‐cadherin was significantly decreased and the expression of N‐cadherin was extremely increased after treatment with 27‐OHC in HCC tissues in vivo, both of which were partly reversed by the upregulation of SULT2A1 (Figure 6C,D). These results consistently proved that SULT2A1 could affect EMT by regulating the level of 27‐OHC.

We then verified the associations between 27‐OHC, SULT2A1 expression, and p65 levels or Twist1 expression in HCC tissues. We found remarkably consistent correlations between 27‐OHC and p‐65 levels, and between 27‐OHC and Twist1 expression. In addition, upregulation of SULT2A1 could partly reverse these changes caused by the addition of 27‐OHC (Figure 6E,F).

We further constructed orthotopic xenograft models by using shSULT2A1 Huh7 cells to investigate the effect of NF‐kB inhibitor in vivo. The shSULT2A1 Huh7 cells treated with 27‐OHC showed a higher incidence of lung metastatic lesions compared to the shNT group, and the NF‐kB inhibitor Bay11‐7082 remarkably reduced the metastasis occurrence (Figure 6G,H). Moreover, the increased status of EMT and the expression of p65 and Twist1, which resulted from 27‐OHC and downregulation of SULT2A1, were significantly inhibited by Bay11‐7082 (Figure 6I,J). We also found a strong correlation between p‐65 and Twist1 expression (Pearson’s *r* = 0.9379, *p* < 0.001) (Figure 6K). Once again, these results confirm that SULT2A1‐dependent alternation of 27‐OHC could affect EMT by regulating Twist1 through the NF‐κB signaling pathway, contributing to HCC metastasis.

### 3.7 Sulfotransferase 2A1 acts as a potential biomarker in predicting HCC clinical outcomes

**FIGURE 7:**
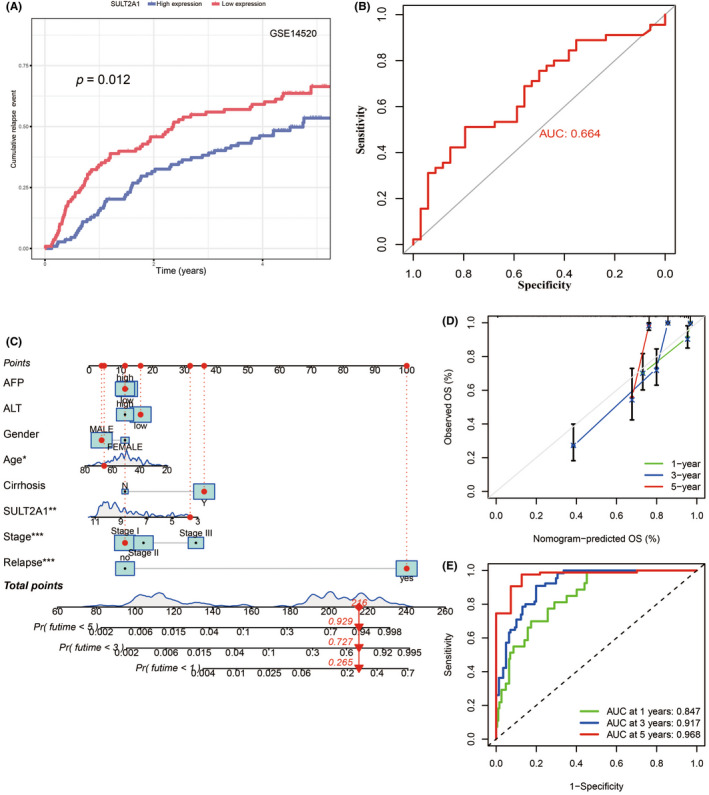
Sulfotransferase family 2A member 1 (SULT2A1) is a potential biomarker in predicting hepatocellular carcinoma (HCC) clinical outcomes. (A) Cumulative relapse event to predict HCC relapse using SULT2A1 based on the Gene Expression Omnibus (GEO) dataset (GSE14520). The median SULT2A1 expression was used as a cut‐off value. (B) Receiver operating characteristic (ROC) curves to predict metastasis (vascular invasion) using SULT2A1 based on the GEO dataset (GSE9843). (C–E) Prognostic nomogram to predict the survival of HCC patients based on The Cancer Genome Atlas Liver Hepatocellular Carcinoma (TCGA‐LIHC) dataset (C). Red circles show clinical characteristics of one specific patient with low expression of SULT2A1 from the GEO dataset (GSE14520). Calibration curves (D) and ROC curves (E) of the nomogram for predicting survival at 1, 3, and 5 years in the GSE14520 dataset are shown. AFP, α‐fetoprotein; ALT, alanine aminotransferase; AUC, area under the ROC curve; OS, overall survival

To establish a clinically applicable method for predicting the prognosis of HCC patients, we attempted to use SULT2A1 as a potential biomarker to predict HCC relapse based on GEO databases (GSE14520). Surprisingly, by analyzing the cumulative relapse event, we discovered an obvious trend in less probability of HCC relapse with high expression of SULT2A1 compared with low expression of SULT2A1 (Figure 7A). We also carried out receiver operating characteristic analyses using SULT2A1 based on a GEO database (GSE9843) and found potential value in predicting HCC metastasis (AUC = 0.664) (Figure 7B).

We established a prognostic nomogram to predict the survival probability at 1, 3, and 5 years based on the GEO databases (GSE14520). Eight independent prognostic parameters, including α‐fetoprotein, alanine aminotransferase, gender, age, cirrhosis, expression of SULT2A1, tumor stage, and relapse, were enrolled in the prediction model (Figure 7C). The calibration plots (Figure 7D) show good consistency between the nomogram prediction and actual observation in terms of the 1‐, 3‐, and 5‐year survival rates in the GSE14520 cohort. The nomogram also showed a favorable predictive ability for the 1‐, 3‐, and 5‐year OS rates, with AUC values of 0.847, 0.917 and 0.968, respectively (Figure 7E). These findings suggest the appreciable reliability of the nomogram. We further assessed the reproducibility of our findings and obtained consistent results in different databases (GSE76427 and The Cancer Genome Atlas Liver Hepatocellular Carcinoma dataset) (Figure S6).

Conclusively, we consider SULT2A1 as a valuable prognostic biomarker. We believe that it might provide new strategies in accurately predicting HCC relapse and metastasis and consequently reduce the mortality caused by HCC.

## 4 DISCUSSION

As revealed by previous researchers, oxysterol is closely related to multiple cancer types, including cancers of the colon, lung, skin, breast, and bile ducts. It has been shown that oxysterol induced cell proliferation and metastasis by promoting oxidative stress and inflammation.^31^ Other studies consider oxysterol a potential predictor of cancer risk.^32^ Here, we discovered a close relationship between the increase of 27‐OHC levels and the metastasis of HCC. In mechanistic terms, SULT2A1‐dependent 27‐OHC changes resulted in the activation of the NF‐κB signaling pathway and promoted EMT by upregulating the transcription factor TWIST1. This finding suggests that the levels of 27‐OHC can be a potential metastatic signal for HCC.

27‐Hydroxycholesterol, an important member of the oxysterol family, was mainly recognized in terms of breast cancer and prostate cancer. As is reported, 27‐OHC behaves as a partial agonist in cellular models of ER+ breast cancer, stimulating their proliferation.^33^ Moreover, 27‐OHC increases cell invasion, migration, and metastasis in prostate cancer.^14^ The impacts of 27‐OHC influencing tumor can be complicated. For one thing, 27‐OHC can induce the expression of pro‐inflammatory cytokines and cause systemic or tissue‐wide local inflammation together with generating EMT, all of which promote tumor initiation, progression, and metastasis.^34^, ^35^, ^36^ In contrast, 27‐OHC exerts an antitumor role by eliciting mitochondrial dysfunction and oxidative stress, leading to the activation of the intrinsic apoptotic pathway.^37^, ^38^ Here, our study identified the role of 27‐OHC in promoting HCC metastasis. However, no difference was found in its effect on tumor proliferation. Interestingly, a previous study reported that 27‐OHC could reverse the effect from cancer promotion to cytotoxicity in HCC by regulating the “switch”‐like molecule GRP75.^39^ This intriguing result might be helpful in understanding our findings and further studies are required to determine the pro‐apoptotic effect of 27‐OHC. We also note that Wang et al. reported that 25‐OHC could promote HCC metastasis, contrary to our findings.^40^ We consider the distinction might be attributed to differences in cell lines (i.e., HepG2 cells).

To date, metabolic changes have been discovered in multiple tumors involving HCC. However, few cases of metastasis‐specific metabolic alternations have been mentioned. In our research, we found that the downregulation of SULT2A1 and increase of 27‐OHC were tightly connected with the metastasis of HCC, indicating the SULT2A1‐dependent increase of 27‐OHC might become a novel hallmark in the metastasis of HCC. Previous researchers have mainly focused on oxysterol promoting tumor metastasis by affecting the tumor immune microenvironment^41^, ^42^ and our study can be a strong complementary proof from the metabolic view in comprehensively understanding the pro‐metastasis effect of oxysterol.

The relationship between SULT and cancer progression has not been well determined. Several early studies discovered the distinct expression patterns of SULT in human cancers,^43^, ^44^ considering it as an independent influencing factor that can act on different signaling pathways.^45^, ^46^ Here, we first linked SULT with HCC metastasis in the metabolic pathway, specifically, that SULT2A1 can affect HCC metastasis by regulating the level of 27‐OHC. Our study has also shown that the expression of SULT2A1 was significantly downregulated, especially in HCC tissues with high metastatic potential. In addition, SULT2A1 has a great capability to predict vascular metastasis and relapse. Therefore, activation of SULT2A1 could be a possible approach for antimetastasis in HCC.

Since it was first identified as an endogenous selective ER modulator and an agonist of LXR,^14^, ^47^ 27‐OHC has been considered to have a close relationship with receptors. By acting on ER, 27‐OHC can increase tumor growth and metastasis in breast cancer,^48^, ^49^ together with functioning as a novel mechanism of resistance to endocrine therapy.^50^ 27‐Hydroxycholesterol can also promote the growth of melanoma cells by activating ERα.^51^ Moreover, LXR is a major target receptor of 27HC, given its purported role in maintaining cholesterol homeostasis by promoting the efflux and reverse transport of cholesterol to the outside of cells.^52^ 27‐Hydroxycholesterol can play a pro‐tumorigenic role by suppressing the expression of various inflammatory genes in macrophages and other immune cells through LXR activation.^53^, ^54^ Interestingly, we note that LXR activation might increase SULT2A1 mRNA levels in human LNCaP prostate cancer cells based on previous research,^55^ and the formation of a range of oxysterol sulfates shows antagonistic effects on LXRs.^56^ The role of LXR in the regulation of SULT expression is still not well determined. Whether LXR functions in the SULT2A1–27‐OHC metabolic axis and in turn impacts on HCC metastasis remains unknown and requires further studies.

In conclusion, our study indicates that the SULT2A1–27‐OHC metabolic axis is a key player in HCC metastasis. Downregulation of SULT2A1 increases the levels of 27‐OHC and promotes the metastasis of HCC by activation of the NF‐κβ signaling pathway together with elevating Twist1 expression and eventually affecting the EMT. Sulfotransferase 2A1 could become a novel prognostic marker for HCC metastasis, and discovering agonists for enhancing SULT2A1 activity could be a potential therapy to suppress the metastasis of HCC.

## DISCLOSURE

The authors have no conflict of interest.

## Supporting information


Figure S1‐S6

Table S1‐S3

Appendix S1
