# Supplementary material for: 27‐Hydroxycholesterol promotes metastasis by SULT2A1‐dependent alteration in hepatocellular carcinoma
Source: Cancer Sci. 2022 Jun 13;113(8):2575–89. doi: 10.1111/cas.15435 (PMC9357618; doi:10.1111/cas.15435)
Supplement: Supplementary file 1 — Figure S1‐S6 [file CAS-113-2575-s003.docx]

Supporting information for

**27-Hydroxycholesterol Promotes Metastasis by SULT2A1-Dependent Alteration in Hepatocellular Carcinoma**

Taochen He^1,2, *^, Baorui Tao^1,2, *^, Chenhe Yi^1,2, *^, Chong Zhang^1,2^, Peng Zhang^1,2^, Weiqing Shao^1^, Yitong Li^1,2^, Zhenmei Chen^1,2^, Lu Lu^1^, Huliang Jia^1^, Wenwei Zhu^1^, Jing Lin^1^, Jinhong Chen^1,2^

*These authors contributed equally to this work.

Authors’ affiliations: ^1^Department of General Surgery, Huashan Hospital, Fudan University, Shanghai, China

^2^Institute of Cancer Metastasis, Fudan University, Shanghai, China

Corresponding author: Jinhong Chen and Jing Lin, Department of General Surgery, Huashan Hospital, Fudan University. 12 Wulumuqi Road (M), Shanghai 200040, China. Tel: +86-21-52887170. E-mail: [jinhongch@hotmail.com](mailto:jinhongch@hotmail.com); Linjingfdu@163.com

**This file includes:**

**Supplementary Figure S1.** The effects of 25-OHC in HCC cells in vitro.

**Supplementary Figure S2.** Expression of SULT2A1, SULT2B1, SULT1E1 in HCC

**Supplementary Figure S3.** Low expression of SULT2A1 is correlated with HCC tissues and the metastatic potential of HCC cell lines

**Supplementary Figure S4.** The expression of SULT2A1 and its correlation with 27-OHC.

**Supplementary Figure S5.** Oxysterols and the effects of EMT in HCC

**Supplementary Figure S6.** The reproducibility of the results shown in Figure 7 have been validated in different cohorts.


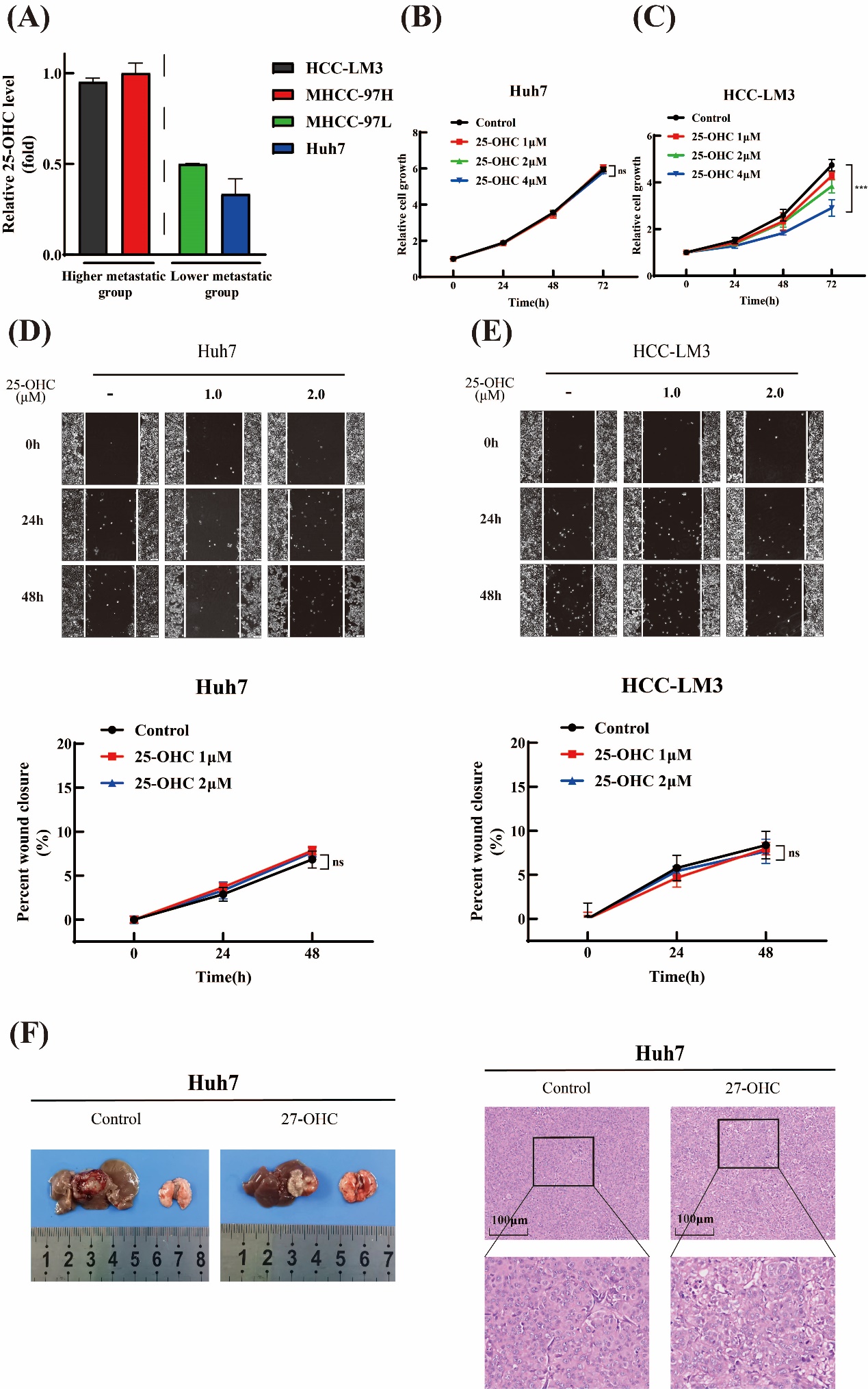
**FIGURE S1**

The effects of 25-OHC in HCC cells *in vitro*. A, Levels of 25-OHC in four common HCC cell lines (HCC-LM3, MHCC-97H, MHCC-97L, Huh7), which were divided into two groups based on their metastatic potential. The levels were determined by enzyme linked sorbent assay (ELSA). Details are described in **Methods**. B-E**,** The effects of 25-OHC on *in vitro* proliferation (B, C; CCK8 assays) and migration (D, E; wound scratch assay) of HCC cells. The relative cell number was calculated as fold change to Time 0h (B, C). Representative photos are shown (D, E). The relative would closure was expressed as the percentage of controls (down of D, E). Representative results from at least three experiments are shown. For (B-E), n=5. Significance was determined by two-way ANOVA (Bonferroni post test). Scale bar, 100μm. F, Representative macroscopic (left) and liver microscopic photos (right) of orthotopic xenograft models of HCC are shown, Scale bar, 100μm.

For Figure S1, each experiment was performed in triplicate. Data are shown as mean ±SD. ^*^p<0.05, ^**^p<0.01, ^***^p<0.001; ns, not significant.


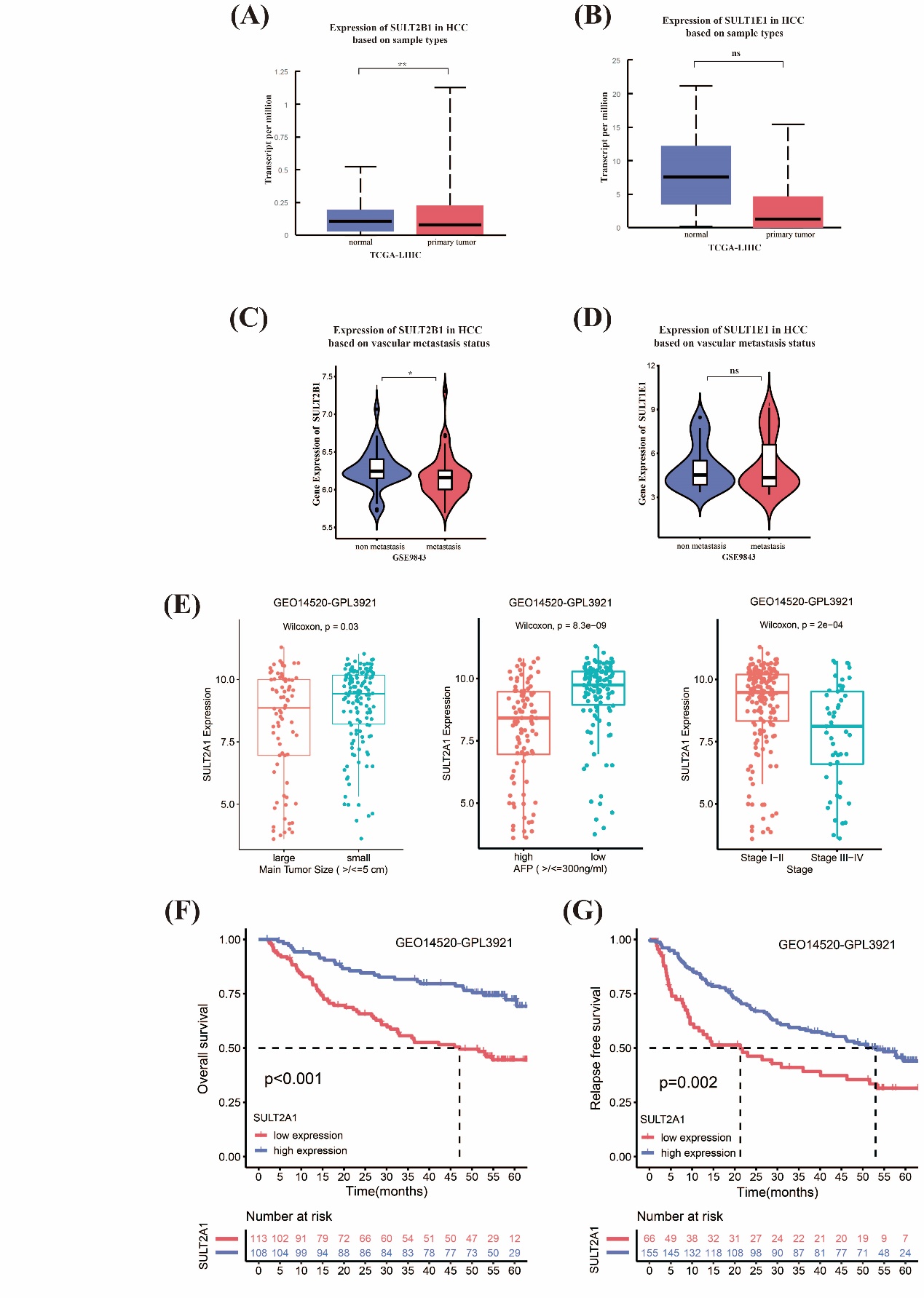


**FIGURE S2**

Expression of SULT2A1, SULT2B1, SULT1E1 in HCC. A, B, The expression of SULT2B1 (A) and SULT1E1 (B) in HCC tissues compared with normal tissues based on TCGA-LIHC database. All data are obtained from The Cancer Genome Atlas (TCGA) dataset (<https://portal.gdc.cancer.gov/>). C, D, The expression of SULT2B1 (C) and SULT1E1 (D) in HCC vascular invasion (metastasis) compared with non-vascular invasion (non-metastasis) tissues based on GEO dataset (GSE9843). All data are obtained from the GEO database (<http://www.ncbi.nih.gov/geo>). E, The expression of SULT2A1 in different tumor sizes, AFP levels and tumor stages of HCC based on GEO dataset (GSE14520). F, G, The prognostic significance of SULT2A1 for HCC patients from GEO dataset (GSE14520) assessed by Kaplan-Meier analysis. Patients with low SULT2A1 expression have poorer overall survival (F) and poorer relapse free survival G) than patients with high SULT2A1 expression.

For Figure S2, significance was determined by Wilcoxon test. ^*^p<0.05, ^**^p<0.01, ^***^p<0.001; ns, not significant.


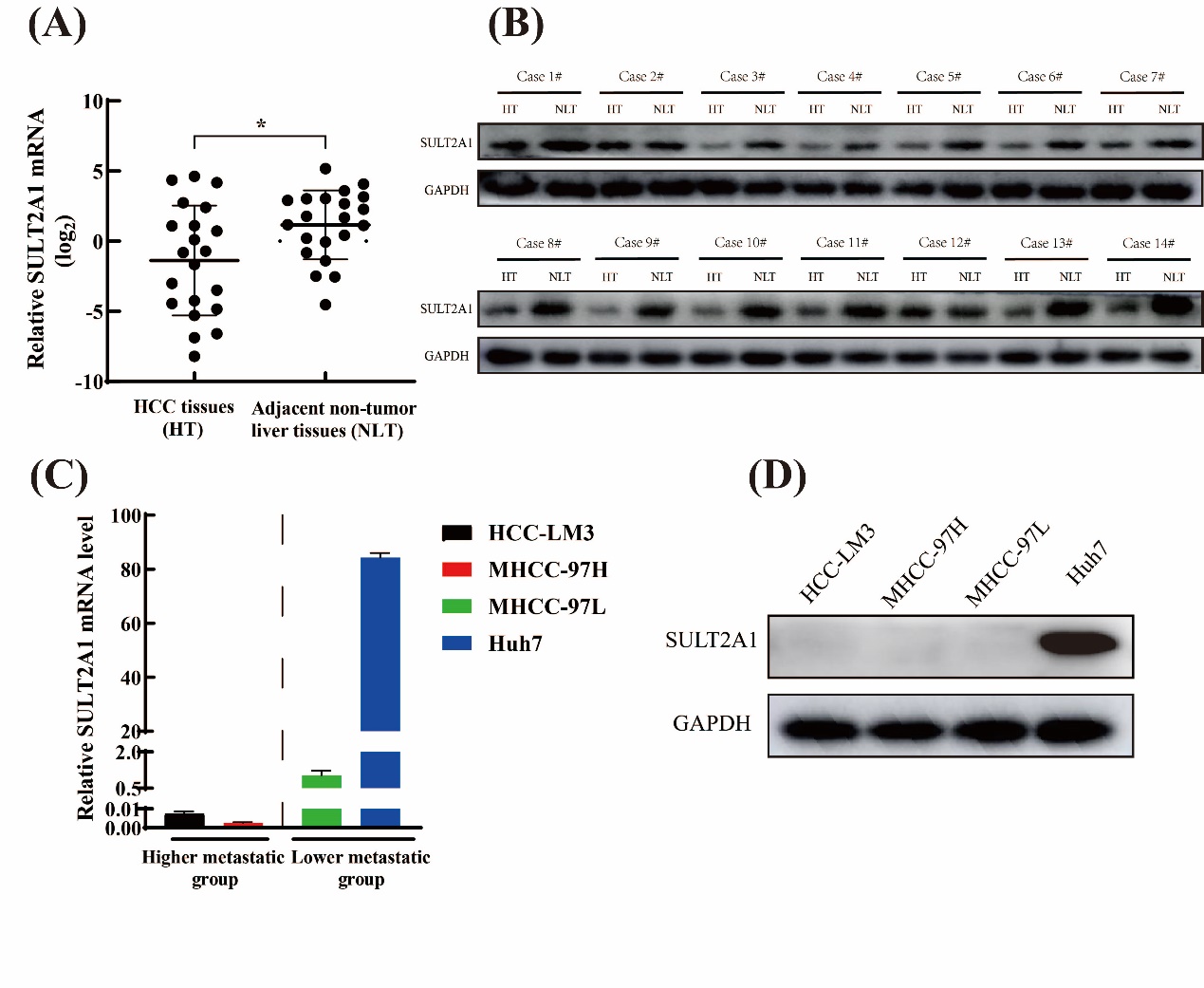
**FIGURE S3**

Low expression of SULT2A1 is correlated with HCC tissues and the metastatic potential of HCC cell lines. A, Quantitative RT-PCR analysis of SULT2A1 mRNA levels in 21 paired samples of human HCC tissues (HT) and matched normal liver tissues (NLT). Data are shown as mean ±SD after log transformation. Significance was determined using the Wilcoxon test. B, immunoblot of SULT2A1 protein in 14 paired samples of human HCC tissues (HT) and matched normal liver tissues (NLT). C, D, Quantitative RT-PCR analysis of SULT2A1 mRNA levels (C) and immunoblot of SULT2A1 protein (D) in HCC cell lines with different metastatic potentials. For (C), data are shown as mean ±SD of triplicate experiments.


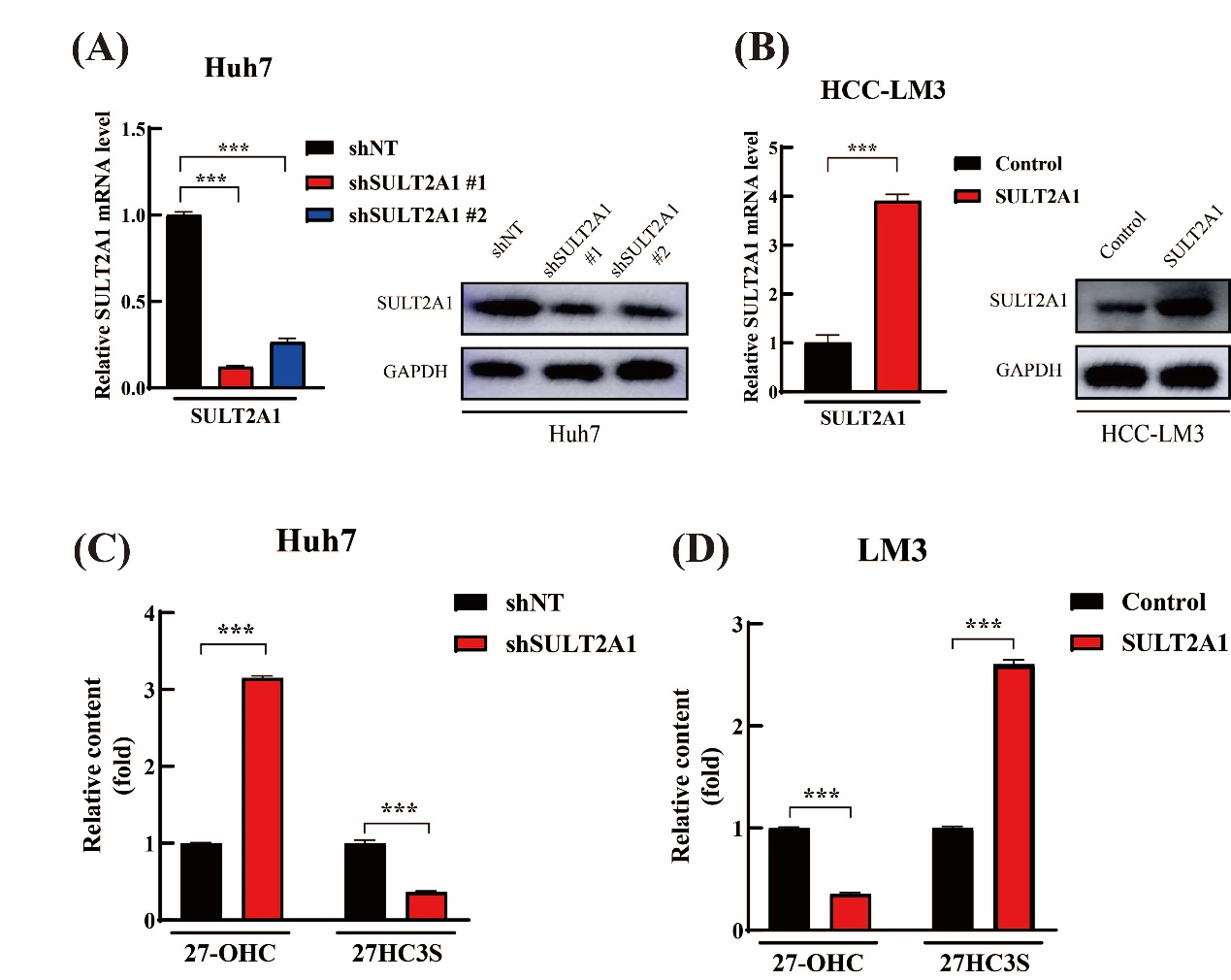


**FIGURE S4**

The expression of SULT2A1 and its correlation with 27-OHC. A, B, Confirmation of SULT2A1 knockdown (shSULT2A1) and overexpression (SULT2A1) in HCC cell lines. Quantitative RT-PCR analysis of SULT2A1 mRNA levels (left of A, B) and immunoblot of SULT2A1 protein (right of A, B) are shown after knockdown of SULT2A1 in Huh7 cells and after overexpression of SULT2A1 in HCC-LM3 cells. C, D, Levels of 27-OHC and 27HC3S (metabolite of 27-OHC by sulfation) in SULT2A1-shRNA stably expressed Huh7 cells (C) or in SULT2A1 stably overexpressed HCC-LM3 cells (D). The levels of 27-OHC were determined by enzyme linked sorbent assay (ELSA) and the levels of 26HC3S were determined by Liquid chromatography-mass spectrometry (LC-MS).

For Figure S4, each experiment was performed in triplicate. Data are shown as mean ±SD. Significance was determined by Student’s t test. ^*^p<0.05, ^**^p<0.01, ^***^p<0.001; ns, not significant.


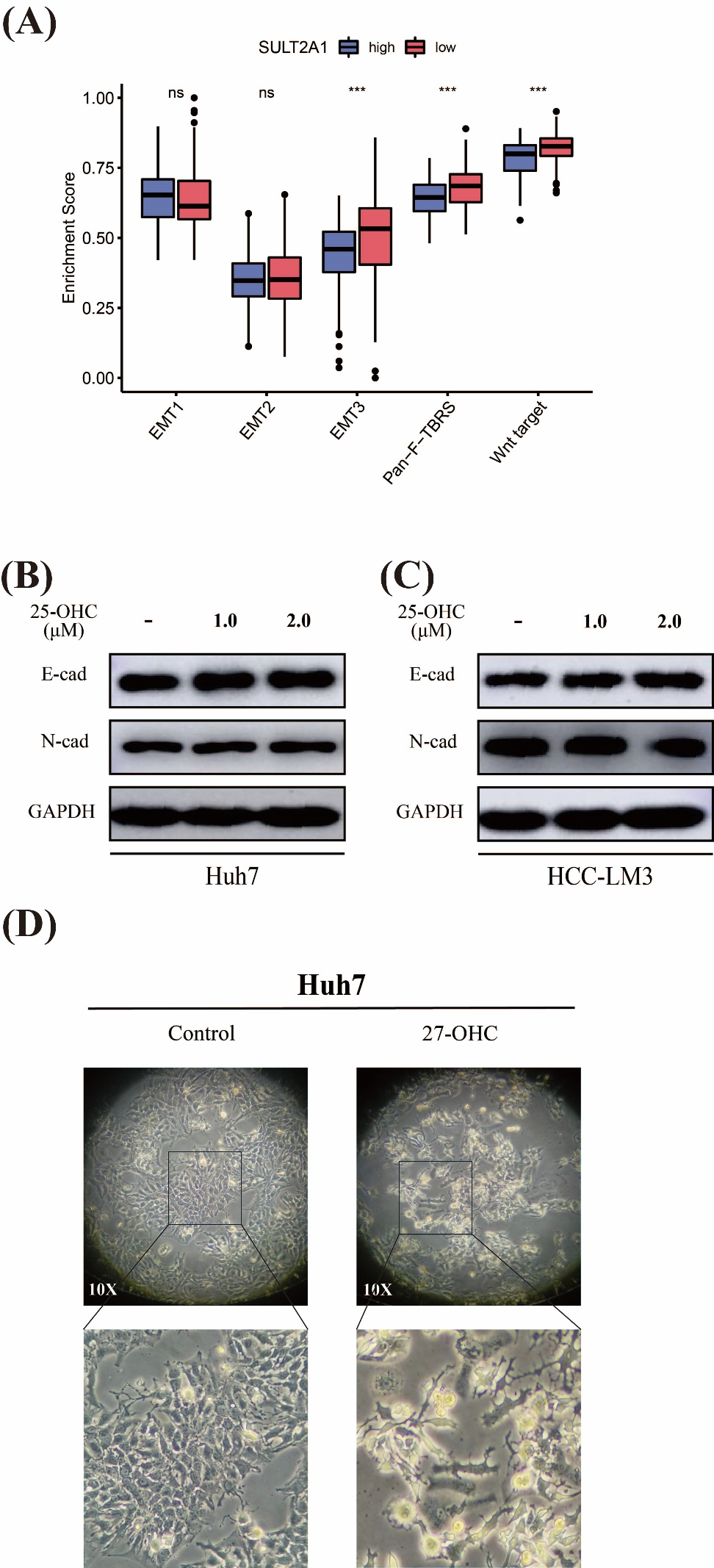


**FIGURE S5**

Oxysterols and the effects of EMT in HCC. A, Enrichment scores of stromal-activation relevant signatures between different expression groups of SULT2A1 based on single sample gene set enrichment analysis (ssGSEA). The median SULT2A1 expression was used as a cutoff value. All data are obtained from GEO dataset (GSE14520). Details are shown in Table S3. B, C, The expression of EMT markers in Huh7 (B) and HCC-LM3 (C) cells treated with different concentration of 25-OHC for 72h was determined by western blotting analysis. Each experiment was performed in triplicate. Representative results are shown. D, Morphological changes in Huh7 cell lines with 27-OHC treatment. Observation was performed under a light microscope.


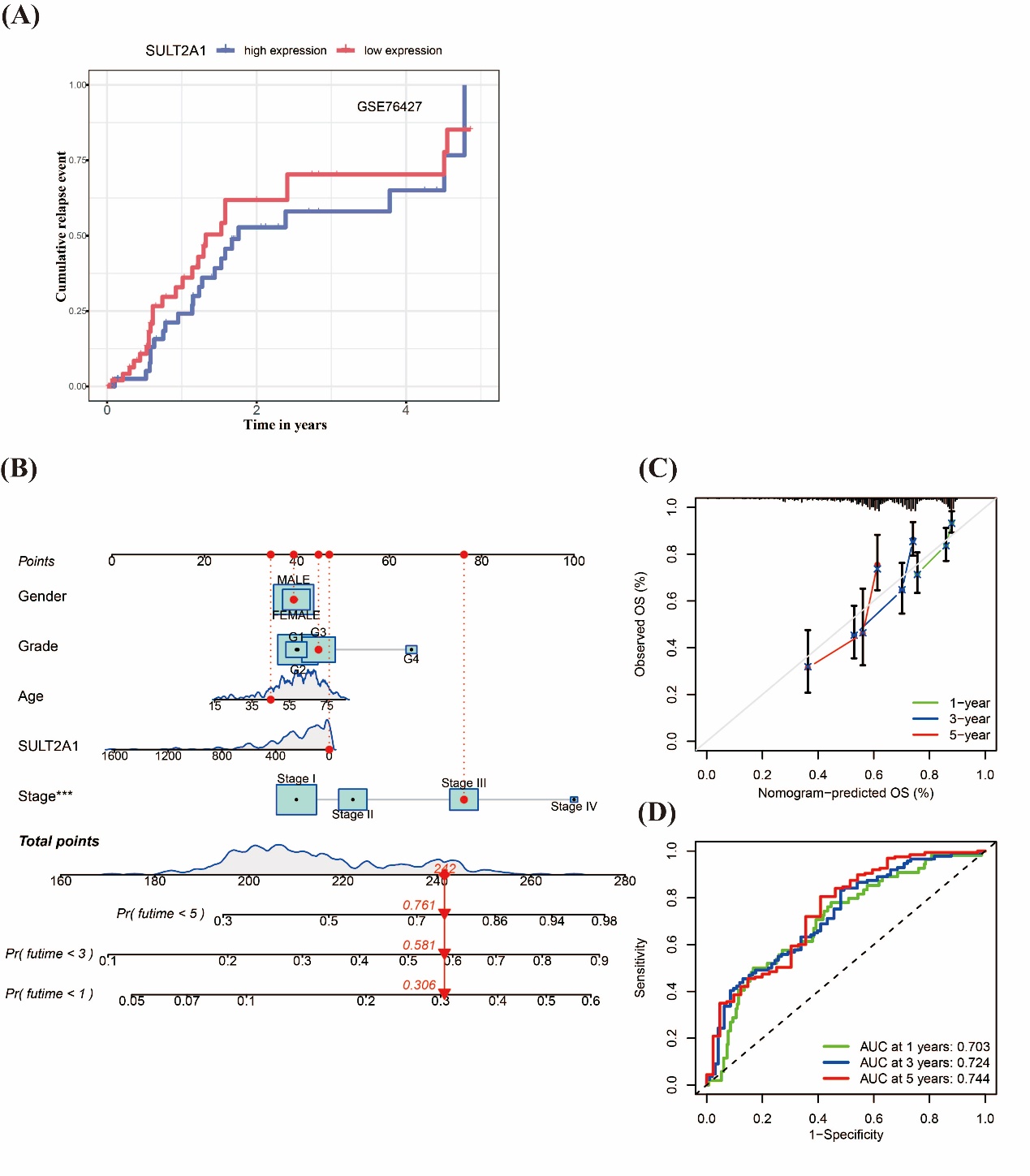


**FIGURE S6**

The reproducibility of the results shown in Figure 7 have been validated in different cohorts. A, Cumulative relapse event to Predict HCC relapse using SULT2A1 based on GEO dataset (GSE76427), The median SULT2A1 expression was used as a cutoff value. B-D, Prognostic nomogram to predict the survival of HCC patients based on the TCGA-LIHC dataset (B). Red points show clinical characteristics of one specific patient with low expression of SULT2A1 from TCGA dataset. Calibration curves (C) and ROC curves (D) of the nomogram for predicting survival at 1, 3, and 5 years in the TCGA dataset are shown.
