# Supplementary material for: 27‐Hydroxycholesterol promotes metastasis by SULT2A1‐dependent alteration in hepatocellular carcinoma
Source: Cancer Sci. 2022 Jun 13;113(8):2575–89. doi: 10.1111/cas.15435 (PMC9357618; doi:10.1111/cas.15435)
Supplement: Supplementary file 2 — Table S1‐S3 [file CAS-113-2575-s001.docx]

Supporting information for

**27-Hydroxycholesterol Promotes Metastasis by SULT2A1-Dependent Alteration in Hepatocellular Carcinoma**

Taochen He^1,2, *^, Baorui Tao^1,2, *^, Chenhe Yi^1,2, *^, Chong Zhang^1,2^, Peng Zhang^1,2^, Weiqing Shao^1^, Yitong Li^1,2^, Zhenmei Chen^1,2^, Lu Lu^1^, Huliang Jia^1^, Wenwei Zhu^1^, Jing Lin^1^, Jinhong Chen^1,2^

*These authors contributed equally to this work.

Authors’ affiliations: ^1^Department of General Surgery, Huashan Hospital, Fudan University, Shanghai, China

^2^Institute of Cancer Metastasis, Fudan University, Shanghai, China

Corresponding author: Jinhong Chen and Jing Lin, Department of General Surgery, Huashan Hospital, Fudan University. 12 Wulumuqi Road (M), Shanghai 200040, China. Tel: +86-21-52887170. E-mail: [jinhongch@hotmail.com](mailto:jinhongch@hotmail.com); Linjingfdu@163.com

**This file includes:**

**Supplementary Table S1**. Contents of different stromal-activation relevant signatures

**Supplementary Table S2**. Detailed data of different stromal-activation relevant signatures based on TCGA datasets

**Supplementary Table S3**. Detailed data of different stromal-activation relevant signatures based on GEO dataset (GSE14520).

**Supplementary Table S1.** Contents of different stromal-activation relevant signatures

| EMT1 | EMT2 | EMT3 | Pan-F-TBRS | Wnt target | Angiogenesis |
| --- | --- | --- | --- | --- | --- |
| na | na | na | na | na | na |
| CLDN3 | AXL | FOXF1 | ACTA2 | EFNB3 | CDH5 |
| CLDN7 | FAP | GATA6 | ACTG2 | MYC | SOX17 |
| CLDN4 | LOXL2 | SOX9 | ADAM12 | TCF12 | SOX18 |
| CDH1 | ROR2 | TWIST1 | ADAM19 | VEGFA | TEK |
| VIM | TAGLN | ZEB1 | CNN1 |  |  |
| TWIST1 | TWIST2 | ZEB2 | COL4A1 |  |  |
| ZEB1 | WNT5A |  | CTGF |  |  |
| ZEB2 |  |  | CTPS1 |  |  |
|  |  |  | FAM101B |  |  |
|  |  |  | FSTL3 |  |  |
|  |  |  | HSPB1 |  |  |
|  |  |  | IGFBP3 |  |  |
|  |  |  | PXDC1 |  |  |
|  |  |  | SEMA7A |  |  |
|  |  |  | SH3PXD2A | |  |
|  |  |  | TAGLN |  |  |
|  |  |  | TGFBI |  |  |
|  |  |  | TNS1 |  |  |
|  |  |  | TPM1 |  |  |

**Supplementary Table S2.** Detailed data of different stromal-activation relevant signatures based on TCGA datasets

| id | EMT1 | EMT2 | EMT3 | Pan-F-TBRS | Wnt target | Angiogenesis |
| --- | --- | --- | --- | --- | --- | --- |
| TCGA-DD-AAE4 | 0.69894 | 0.466381 | 0.386778 | 0.773639 | 0.545967 | 0.736296 |
| TCGA-CC-A3M9 | 0.543965 | 0.725269 | 0.554513 | 0.969454 | 0.684144 | 0.720543 |
| TCGA-DD-A4NI | 0.672591 | 0.42635 | 0.487146 | 0.809794 | 0.690947 | 0.702432 |
| TCGA-G3-AAV3 | 0.671727 | 0.507172 | 0.422989 | 0.812144 | 0.67716 | 0.633756 |
| TCGA-UB-A7MA | 0.708282 | 0.403728 | 0.44436 | 0.808129 | 0.772695 | 0.479846 |
| TCGA-UB-A7MF | 0.743447 | 0.234265 | 0.395645 | 0.692239 | 0.500458 | 0.482092 |
| TCGA-BC-A112 | 0.846399 | 0.604817 | 0.590805 | 0.882379 | 0.795907 | 0.64852 |
| TCGA-BC-A10U | 0.684157 | 0.250261 | 0.341806 | 0.793466 | 0.555606 | 0.114827 |
| TCGA-BC-A5W4 | 0.6902 | 0.121519 | 0.137884 | 0.658639 | 0.534964 | 0.247368 |
| TCGA-FV-A3I1 | 0.823368 | 0.544545 | 0.525118 | 0.80469 | 0.65762 | 0.663695 |
| TCGA-CC-A123 | 0.585309 | 0.322187 | 0.47118 | 0.760507 | 0.653385 | 0.529077 |
| TCGA-WQ-A9G7 | 0.75737 | 0.302195 | 0.34657 | 0.785919 | 0.649992 | 0.492664 |
| TCGA-ED-A66X | 0.865035 | 0.642648 | 0.573758 | 0.923921 | 0.769203 | 0.697492 |
| TCGA-DD-A1EK | 0.612108 | 0.433533 | 0.180683 | 0.857079 | 0.624077 | 0.584594 |
| TCGA-DD-AADM | 0.734651 | 0.44144 | 0.383341 | 0.81798 | 0.575529 | 0.619998 |
| TCGA-MR-A8JO | 0.645303 | 0.669967 | 0.629811 | 0.94677 | 0.768701 | 0.924501 |
| TCGA-QA-A7B7 | 0.517114 | 0.068356 | 0.113875 | 0.684257 | 0.500038 | 0.443421 |
| TCGA-CC-A8HT | 0.664937 | 0.284168 | 0.418293 | 0.805493 | 0.614817 | 0.405116 |
| TCGA-FV-A4ZP | 0.720947 | 0.284217 | 0.396865 | 0.764998 | 0.524171 | 0.373787 |
| TCGA-DD-A4NJ | 0.742998 | 0.479256 | 0.396347 | 0.803877 | 0.576592 | 0.564682 |
| TCGA-CC-5258 | 0.553167 | 0.444582 | 0.497066 | 0.841002 | 0.717331 | 0.655582 |
| TCGA-EP-A2KB | 0.591352 | 0.183951 | 0.438261 | 0.731914 | 0.676031 | 0.499321 |
| TCGA-G3-A25X | 0.701028 | 0.562405 | 0.526113 | 0.858797 | 0.730263 | 0.633328 |
| TCGA-DD-AAD1 | 0.742865 | 0.566719 | 0.621099 | 0.907309 | 0.802742 | 0.801039 |
| TCGA-G3-A3CK | 0.630995 | 0.386496 | 0.427708 | 0.777232 | 0.59953 | 0.533105 |
| TCGA-DD-AACA | 0.584023 | 0.305026 | 0.361029 | 0.781875 | 0.736485 | 0.659289 |
| TCGA-5C-A9VH | 0.817591 | 0.474731 | 0.519835 | 0.861597 | 0.718599 | 0.826388 |
| TCGA-FV-A23B | 0.568442 | 0.215012 | 0.497882 | 0.83647 | 0.754967 | 0.653834 |
| TCGA-DD-AADR | 0.65672 | 0.280088 | 0.301501 | 0.786974 | 0.283488 | 0.69811 |
| TCGA-DD-A4NQ | 0.683027 | 0.374424 | 0.414536 | 0.755705 | 0.587959 | 0.279993 |
| TCGA-G3-A5SM | 0.735103 | 0.557574 | 0.522299 | 0.824517 | 0.652832 | 0.737886 |
| TCGA-MI-A75E | 0.757937 | 0.539181 | 0.511458 | 0.855347 | 0.676467 | 0.76079 |
| TCGA-DD-A11D | 0.692676 | 0.338655 | 0.507915 | 0.79456 | 0.67733 | 0.63785 |
| TCGA-CC-5262 | 0.681694 | 0.637896 | 0.603952 | 0.911073 | 0.699868 | 0.803909 |
| TCGA-2Y-A9GY | 0.646514 | 0.171962 | 0.373896 | 0.70833 | 0.608697 | 0.1694 |
| TCGA-ES-A2HT | 0.653824 | 0.467791 | 0.427765 | 0.815511 | 0.636757 | 0.741607 |
| TCGA-DD-AAW0 | 0.742138 | 0.569377 | 0.565468 | 0.839686 | 0.7411 | 0.732283 |
| TCGA-G3-A3CI | 0.593644 | 0.303177 | 0.434938 | 0.782585 | 0.686305 | 0.651916 |
| TCGA-DD-AAVV | 0.781009 | 0.530781 | 0.539764 | 0.872621 | 0.71898 | 0.779516 |
| TCGA-DD-A39Y | 0.725349 | 0.350403 | 0.39394 | 0.741912 | 0.632694 | 0.367991 |
| TCGA-DD-A1EB | 0.663099 | 0.269488 | 0.381802 | 0.73941 | 0.63442 | 0.745665 |
| TCGA-DD-AAD6 | 0.649956 | 0.108579 | 0.296959 | 0.634071 | 0.696699 | 0.327603 |
| TCGA-ED-A4XI | 0.772537 | 0.559434 | 0.545601 | 0.824246 | 0.745576 | 0.768893 |
| TCGA-HP-A5MZ | 0.792997 | 0.654607 | 0.620558 | 0.862768 | 0.713586 | 0.83377 |
| TCGA-DD-AAVU | 0.578843 | 0.183425 | 0.199203 | 0.788918 | 0.589379 | 0.430185 |
| TCGA-DD-A4NO | 0.748402 | 0.491688 | 0.49624 | 0.829474 | 0.705455 | 0.67609 |
| TCGA-BC-A10W | 0.817895 | 0.411139 | 0.561242 | 0.848513 | 0.745613 | 0.593567 |
| TCGA-LG-A9QC | 0.665787 | 0.282724 | 0.398996 | 0.856634 | 0.665655 | 0.675766 |
| TCGA-DD-A11C | 0.604865 | 0.608737 | 0.36933 | 0.854636 | 0.646928 | 0.821538 |
| TCGA-DD-AADB | 0.482043 | 0.261823 | 0.465362 | 0.785374 | 0.654418 | 0.557107 |
| TCGA-DD-AAVZ | 0.749521 | 0.413463 | 0.439352 | 0.790534 | 0.709928 | 0.682191 |
| TCGA-BC-A216 | 0.797811 | 0.182828 | 0.556582 | 0.798684 | 0.649799 | 0.158373 |
| TCGA-DD-A1EF | 0.651479 | 0.449182 | 0.55946 | 0.737004 | 0.757493 | 0.567701 |
| TCGA-DD-AADL | 0.675218 | 0.216086 | 0.409713 | 0.740014 | 0.577273 | 0.524953 |
| TCGA-KR-A7K8 | 0.649751 | 0.467376 | 0.348144 | 0.873379 | 0.655889 | 0.663452 |
| TCGA-ED-A7PX | 0.838104 | 0.635354 | 0.594369 | 0.930565 | 0.730835 | 0.687454 |
| TCGA-DD-AADK | 0.67708 | 0.564396 | 0.508514 | 0.8307 | 0.622507 | 0.712499 |
| TCGA-BW-A5NO | 0.69967 | 0.354611 | 0.318677 | 0.744078 | 0.470322 | 0.468659 |
| TCGA-DD-A39W | 0.450468 | 0.408796 | 0.408628 | 0.73559 | 0.632343 | 0.622407 |
| TCGA-GJ-A9DB | 0.789325 | 0.508174 | 0.526662 | 0.836057 | 0.620501 | 0.615759 |
| TCGA-DD-A1EA | 0.56538 | 0.216883 | 0.379061 | 0.697732 | 0.621282 | 0.568055 |
| TCGA-UB-A7MD | 0.763374 | 0.449918 | 0.435581 | 0.815439 | 0.662543 | 0.752021 |
| TCGA-ES-A2HS | 0.634064 | 0.442455 | 0.483312 | 0.816682 | 0.657079 | 0.739722 |
| TCGA-G3-A3CJ | 0.493743 | 0.318729 | 0.504074 | 0.792568 | 0.610645 | 0.663629 |
| TCGA-FV-A3R2 | 0.649448 | 0.306509 | 0.465163 | 0.752412 | 0.69426 | 0.495534 |
| TCGA-BW-A5NQ | 0.659714 | 0.31012 | 0.395056 | 0.691337 | 0.609998 | 0.522104 |
| TCGA-2Y-A9GZ | 0.574742 | 0.245776 | 0.080904 | 0.737642 | 0.620818 | 0.710917 |
| TCGA-4R-AA8I | 0.612848 | 0.288716 | 0.315093 | 0.774659 | 0.633203 | 0.567583 |
| TCGA-DD-A4NG | 0.571847 | 0.281923 | 0.441995 | 0.821449 | 0.728429 | 0.591992 |
| TCGA-RC-A6M5 | 0.697513 | 0.617484 | 0.495911 | 0.890575 | 0.787227 | 0.868414 |
| TCGA-2Y-A9H0 | 0.603401 | 0.213711 | 0.286851 | 0.687781 | 0.47369 | 0.286324 |
| TCGA-DD-A11B | 0.579911 | 0.435356 | 0.486459 | 0.757006 | 0.539459 | 0.598394 |
| TCGA-G3-A25V | 0.71093 | 0.589251 | 0.560868 | 0.8443 | 0.777255 | 0.708863 |
| TCGA-5R-AA1C | 0.515683 | 0.228388 | 0.298118 | 0.668687 | 0.642873 | 0.444966 |
| TCGA-ED-A8O6 | 0.787325 | 0.484334 | 0.479004 | 0.85601 | 0.73622 | 0.519347 |
| TCGA-DD-AADD | 0.684893 | 0.089925 | 0.357849 | 0.657096 | 0.622268 | 0.458206 |
| TCGA-BC-A10S | 0.738595 | 0.57847 | 0.526763 | 0.87041 | 0.748357 | 0.782393 |
| TCGA-CC-A9FV | 0.486746 | 0.586163 | 0.648869 | 0.797829 | 0.527428 | 0.671017 |
| TCGA-DD-AAEB | 0.613319 | 0.371678 | 0.284727 | 0.730497 | 0.595479 | 0.667243 |
| TCGA-WX-AA44 | 0.778788 | 0.425706 | 0.493953 | 0.796717 | 0.599532 | 0.51552 |
| TCGA-PD-A5DF | 0.839831 | 0.546623 | 0.551879 | 0.871538 | 0.630386 | 0.6423 |
| TCGA-YA-A8S7 | 0.66465 | 0.559713 | 0.485953 | 0.802049 | 0.700638 | 0.536607 |
| TCGA-5R-AA1D | 0.755308 | 0.721641 | 0.645616 | 0.870062 | 0.816416 | 1 |
| TCGA-DD-AACH | 0.741931 | 0.408947 | 0.485161 | 0.80096 | 0.716259 | 0.625878 |
| TCGA-CC-A8HV | 0.776377 | 0.554376 | 0.417356 | 0.805781 | 0.446657 | 0.391859 |
| TCGA-BW-A5NP | 0.595406 | 0.416545 | 0.44663 | 0.763039 | 0.702014 | 0.532815 |
| TCGA-G3-AAV0 | 0.655266 | 0.32763 | 0.427964 | 0.741405 | 0.48119 | 0.664315 |
| TCGA-DD-AAED | 0.619602 | 0.096937 | 0.156305 | 0.712431 | 0.520059 | 0.105014 |
| TCGA-DD-A73F | 0.63021 | 0.367875 | 0.281091 | 0.79421 | 0.544705 | 0.614576 |
| TCGA-DD-A4NN | 0.8039 | 0.42857 | 0.544031 | 0.840004 | 0.689141 | 0.67578 |
| TCGA-DD-AAEI | 0.68282 | 0.366772 | 0.439986 | 0.726731 | 0.680955 | 0.641923 |
| TCGA-UB-AA0V | 0.794491 | 0.52005 | 0.613361 | 0.865768 | 0.788488 | 0.794823 |
| TCGA-DD-AADO | 0.651332 | 0.1909 | 0.361779 | 0.767973 | 0.602338 | 0.424738 |
| TCGA-DD-AAVY | 0.526051 | 0.242316 | 0.251424 | 0.700485 | 0.679446 | 0.590435 |
| TCGA-FV-A3R3 | 0.816749 | 0.59116 | 0.581656 | 0.833388 | 0.776358 | 0.879756 |
| TCGA-DD-A4NS | 0.848687 | 0.606404 | 0.681089 | 0.943959 | 0.826683 | 0.8308 |
| TCGA-MI-A75H | 0.541807 | 0.370842 | 0.43508 | 0.810312 | 0.649948 | 0.756128 |
| TCGA-UB-A7MC | 0.714262 | 0.130882 | 0.286037 | 0.681016 | 0.636927 | 0.508045 |
| TCGA-FV-A4ZQ | 0.548122 | 0.280185 | 0.294555 | 0.674569 | 0.689254 | 0.33427 |
| TCGA-DD-AAEG | 0.766013 | 0.423288 | 0.431062 | 0.807103 | 0.663512 | 0.719791 |
| TCGA-DD-AAD5 | 0.630009 | 0.246773 | 0.382009 | 0.754074 | 0.709279 | 0.432507 |
| TCGA-K7-A5RG | 0.791868 | 0.434579 | 0.456964 | 0.732683 | 0.570797 | 0.607628 |
| TCGA-XR-A8TD | 0.706838 | 0.617741 | 0.50203 | 0.90303 | 0.685966 | 0.713215 |
| TCGA-DD-AACY | 0.702932 | 0.339687 | 0.483256 | 0.826449 | 0.725537 | 0.671361 |
| TCGA-CC-A9FU | 0.485363 | 0.110784 | 0.327487 | 0.738403 | 0.648363 | 0.346644 |
| TCGA-KR-A7K7 | 0.627544 | 0.120262 | 0.294218 | 0.683696 | 0.591426 | 0.463224 |
| TCGA-G3-A25Y | 0.700387 | 0.56526 | 0.569714 | 0.857489 | 0.76823 | 0.707337 |
| TCGA-K7-AAU7 | 0.884367 | 0.587649 | 0.67441 | 0.896241 | 0.764675 | 0.483479 |
| TCGA-BC-A3KF | 0.676818 | 0.310523 | 0.27388 | 0.748813 | 0.61079 | 0.601018 |
| TCGA-BC-A10Z | 0.65805 | 0.052535 | 0.092783 | 0.661238 | 0.486526 | 0.466979 |
| TCGA-UB-AA0U | 0.773261 | 0.560135 | 0.534789 | 0.861244 | 0.730579 | 0.704301 |
| TCGA-DD-AAEE | 0.596189 | 0.316785 | 0.342484 | 0.76619 | 0.562818 | 0.694426 |
| TCGA-ZP-A9D0 | 0.650096 | 0.365355 | 0.469422 | 0.791099 | 0.726469 | 0.642572 |
| TCGA-DD-A1EG | 0.821348 | 0.562889 | 0.583298 | 0.820395 | 0.682819 | 0.679863 |
| TCGA-CC-A7IL | 0.598753 | 0 | 0.313671 | 0.733345 | 0.676268 | 0.513707 |
| TCGA-RC-A6M4 | 0.410887 | 0.295193 | 0.219356 | 0.739161 | 0.618134 | 0.442432 |
| TCGA-ED-A7XP | 0.772719 | 0.451916 | 0.466513 | 0.814038 | 0.665424 | 0.708367 |
| TCGA-2Y-A9HA | 0.718445 | 0.279836 | 0.439514 | 0.722305 | 0.68729 | 0.556289 |
| TCGA-DD-AAE2 | 0.711625 | 0.445567 | 0.292147 | 0.770607 | 0.626145 | 0.733982 |
| TCGA-DD-AADF | 0.597626 | 0.255137 | 0.29987 | 0.740527 | 0.405506 | 0.483942 |
| TCGA-DD-AACW | 0.525371 | 0.186725 | 0.326788 | 0.705378 | 0.623123 | 0.572096 |
| TCGA-MI-A75I | 0.618341 | 0.329288 | 0.377623 | 0.772393 | 0.599946 | 0.527737 |
| TCGA-FV-A3I0 | 0.833433 | 0.684019 | 0.53852 | 0.90197 | 0.791471 | 0.499888 |
| TCGA-2Y-A9H1 | 0.753048 | 0.251752 | 0.399038 | 0.750319 | 0.214166 | 0.651977 |
| TCGA-CC-A8HU | 0.740238 | 0.37779 | 0.398281 | 0.810137 | 0.536832 | 0.383057 |
| TCGA-DD-AAD2 | 0.761222 | 0.642444 | 0.547452 | 0.90483 | 0.718137 | 0.727672 |
| TCGA-BC-A10R | 0.637777 | 0.579031 | 0.522877 | 0.832788 | 0.740024 | 0.811147 |
| TCGA-G3-A5SL | 0.584177 | 0.544776 | 0.462923 | 0.83788 | 0.706216 | 0.647241 |
| TCGA-CC-A1HT | 0.744137 | 0.526117 | 0.561321 | 0.872488 | 0.619294 | 0.588218 |
| TCGA-2Y-A9GV | 0.643003 | 0.413723 | 0.305647 | 0.764721 | 0.645252 | 0.734318 |
| TCGA-ED-A7XO | 0.741228 | 0.500379 | 0.413909 | 0.839781 | 0.692382 | 0.766867 |
| TCGA-CC-A5UC | 0.663697 | 0.466478 | 0.517266 | 0.88464 | 0.633779 | 0.751818 |
| TCGA-DD-AAVQ | 0.784522 | 0.483801 | 0.527732 | 0.818256 | 0.58454 | 0.668852 |
| TCGA-BD-A3EP | 0.710513 | 0.52248 | 0.508482 | 0.876327 | 0.70786 | 0.800292 |
| TCGA-DD-A39V | 0.582557 | 0.34863 | 0.41144 | 0.789691 | 0.564983 | 0.61255 |
| TCGA-DD-A3A1 | 0.732916 | 0.160121 | 0.349119 | 0.728493 | 0.591227 | 0.393633 |
| TCGA-DD-A4NR | 0.823996 | 0.573716 | 0.42668 | 0.917055 | 0.632346 | 0.593688 |
| TCGA-DD-AAC9 | 0.785642 | 0.551825 | 0.514009 | 0.855769 | 0.691109 | 0.742583 |
| TCGA-CC-5259 | 0.66478 | 0.194308 | 0.46041 | 0.706479 | 0.174087 | 0.516906 |
| TCGA-DD-AADC | 0.668023 | 0.274496 | 0.478515 | 0.79599 | 0.602252 | 0.601525 |
| TCGA-ZS-A9CF | 0.746876 | 0.247578 | 0.308348 | 0.7653 | 0.601165 | 0.557362 |
| TCGA-NI-A8LF | 0.797873 | 0.467174 | 0.499668 | 0.862057 | 0.692433 | 0.802655 |
| TCGA-DD-A39Z | 0.474023 | 0.264585 | 0.237289 | 0.808097 | 0.520084 | 0.624566 |
| TCGA-G3-A7M5 | 0.622117 | 0.213459 | 0.217548 | 0.75151 | 0.654015 | 0.553682 |
| TCGA-MI-A75G | 0.654641 | 0.32473 | 0.344936 | 0.796702 | 0.614039 | 0.575909 |
| TCGA-CC-A7IK | 0.718856 | 0.411803 | 0.403417 | 0.665076 | 0.665662 | 0.553331 |
| TCGA-G3-A5SJ | 0.829571 | 0.390006 | 0.597054 | 0.793146 | 0.717669 | 0.457589 |
| TCGA-ZS-A9CE | 0.603338 | 0.250119 | 0.30575 | 0.720588 | 0.624204 | 0.566956 |
| TCGA-DD-A73B | 0.599723 | 0.244135 | 0.279153 | 0.663387 | 0.514565 | 0.48512 |
| TCGA-RC-A7SB | 0.628917 | 0.334482 | 0.401057 | 0.769345 | 0.554866 | 0.56947 |
| TCGA-DD-AADN | 0.539571 | 0.18435 | 0.086897 | 0.610663 | 0.236327 | 0.335092 |
| TCGA-CC-A5UE | 0.579362 | 0.033455 | 0.157541 | 0.681073 | 0.574059 | 0.366773 |
| TCGA-DD-AACZ | 0.531321 | 0.284453 | 0.416975 | 0.752631 | 0.655349 | 0.521085 |
| TCGA-DD-A3A3 | 0.506281 | 0.295091 | 0.087024 | 0.795837 | 0.592092 | 0.537086 |
| TCGA-2Y-A9H8 | 0.775232 | 0.386331 | 0.402454 | 0.841719 | 0.445613 | 0.461316 |
| TCGA-DD-AAEK | 0.61505 | 0.477047 | 0.47572 | 0.828337 | 0.737054 | 0.663512 |
| TCGA-DD-A4ND | 0.799552 | 0.623589 | 0.596411 | 0.951015 | 0.731412 | 0.775835 |
| TCGA-2Y-A9H7 | 0.557894 | 0.218257 | 0.273247 | 0.764294 | 0.628366 | 0.662295 |
| TCGA-RC-A7S9 | 0.676497 | 0.188715 | 0.286228 | 0.714913 | 0.431635 | 0.542569 |
| TCGA-G3-A25S | 0.670619 | 0.081346 | 0.149957 | 0.705706 | 0.50801 | 0.346073 |
| TCGA-BC-A217 | 0.626746 | 0.12209 | 0.301688 | 0.5957 | 0.350491 | 0.102057 |
| TCGA-DD-AAW2 | 0.694665 | 0.346976 | 0.444029 | 0.805991 | 0.729559 | 0.705063 |
| TCGA-2Y-A9H6 | 0.671164 | 0.469456 | 0.472149 | 0.848338 | 0.716948 | 0.774185 |
| TCGA-2Y-A9GW | 0.830978 | 0.541407 | 0.591134 | 0.837149 | 0.699602 | 0.688544 |
| TCGA-DD-A73D | 0.429182 | 0.108945 | 0.086235 | 0.665236 | 0.683409 | 0.490703 |
| TCGA-DD-A115 | 0.767275 | 0.486063 | 0.4159 | 0.778143 | 0.663756 | 0.709554 |
| TCGA-XR-A8TF | 0.601199 | 0.14276 | 0.401441 | 0.732356 | 0.676228 | 0.560051 |
| TCGA-DD-AADU | 0.70934 | 0.306175 | 0.4453 | 0.690719 | 0.613343 | 0.486254 |
| TCGA-DD-AAE1 | 0.462955 | 0.162486 | 0.275603 | 0.691719 | 0.613414 | 0.601854 |
| TCGA-2Y-A9HB | 0.620132 | 0.339452 | 0.273599 | 0.790184 | 0.607623 | 0.68282 |
| TCGA-BD-A2L6 | 0.747406 | 0.236055 | 0.310282 | 0.764159 | 0.506835 | 0.634884 |
| TCGA-DD-A3A9 | 0.646701 | 0.54389 | 0.543885 | 0.871641 | 0.708798 | 0.813957 |
| TCGA-KR-A7K2 | 0.790662 | 0.410675 | 0.49975 | 0.792862 | 0.596577 | 0.678066 |
| TCGA-ED-A66Y | 0.4909 | 0.587863 | 0.519324 | 0.83659 | 0.779975 | 0.511278 |
| TCGA-DD-AAE3 | 0.798455 | 0.418182 | 0.450827 | 0.80998 | 0.676276 | 0.754951 |
| TCGA-G3-AAV6 | 0.619254 | 0.469897 | 0.467088 | 0.751909 | 0.605814 | 0.506511 |
| TCGA-ED-A97K | 0.827474 | 0.552906 | 0.573479 | 0.828262 | 0.722838 | 0.532851 |
| TCGA-DD-AAVW | 0.861582 | 0.593657 | 0.670707 | 0.838771 | 0.754143 | 0.859086 |
| TCGA-EP-A3RK | 0.605926 | 0.436772 | 0.452159 | 0.786291 | 0.656316 | 0.582859 |
| TCGA-FV-A496 | 0.540554 | 0.271774 | 0.177188 | 0.718557 | 0.614554 | 0.57568 |
| TCGA-CC-A3MB | 0.668881 | 0.252419 | 0.483992 | 0.716782 | 0.72583 | 0.582864 |
| TCGA-CC-A7IF | 0.629041 | 0.380247 | 0.362286 | 0.796811 | 0.618007 | 0.625436 |
| TCGA-ZS-A9CD | 0.697569 | 0.542737 | 0.540253 | 0.851623 | 0.707164 | 0.737039 |
| TCGA-G3-A7M6 | 0.743104 | 0.436198 | 0.476745 | 0.765437 | 0.772735 | 0.668609 |
| TCGA-DD-A1ED | 0.702455 | 0.563508 | 0.540293 | 0.808519 | 0.754505 | 0.745785 |
| TCGA-EP-A12J | 0.636661 | 0.265554 | 0.439414 | 0.723887 | 0.641622 | 0.636313 |
| TCGA-DD-A1EJ | 0.539103 | 0.288958 | 0.285188 | 0.713868 | 0.681448 | 0.35296 |
| TCGA-G3-AAV2 | 0.407052 | 0.27521 | 0.143804 | 0.755375 | 0.591255 | 0.715102 |
| TCGA-DD-AADY | 0.693133 | 0.249327 | 0.58297 | 0.774057 | 0.714786 | 0.750051 |
| TCGA-CC-5263 | 0.704186 | 0.317013 | 0.46549 | 0.727892 | 0.76143 | 0.458453 |
| TCGA-DD-AADP | 0.732753 | 0.390816 | 0.536261 | 0.859607 | 0.719293 | 0.68468 |
| TCGA-BC-A69I | 0.643606 | 0.516954 | 0.481597 | 0.838452 | 0.647086 | 0.745826 |
| TCGA-5C-A9VG | 0.661771 | 0.493102 | 0.469903 | 0.850323 | 0.70334 | 0.745192 |
| TCGA-DD-A1EE | 0.711428 | 0.276927 | 0.388777 | 0.784898 | 0.678836 | 0.54997 |
| TCGA-BC-A110 | 0.786024 | 0.625333 | 0.601887 | 0.90368 | 0.789987 | 0.822152 |
| TCGA-GJ-A3OU | 0.744375 | 0.650962 | 0.536379 | 0.850368 | 0.69905 | 0.822512 |
| TCGA-G3-A25U | 0.634554 | 0.182372 | 0.343203 | 0.732375 | 0.666928 | 0.630644 |
| TCGA-DD-A4NL | 0.758674 | 0.463652 | 0.607777 | 0.800759 | 0.785167 | 0.797803 |
| TCGA-BC-A10Q | 0.843622 | 0.55763 | 0.503174 | 0.84506 | 0.856114 | 0.508166 |
| TCGA-2Y-A9GU | 0.715126 | 0.16194 | 0.426104 | 0.770234 | 0.566935 | 0.601821 |
| TCGA-ZP-A9CV | 0.770542 | 0.45791 | 0.561732 | 0.822447 | 0.66456 | 0.653435 |
| TCGA-DD-A113 | 0.772999 | 0.408595 | 0.445048 | 0.799768 | 0.685705 | 0.661296 |
| TCGA-2Y-A9GT | 0.676736 | 0.422633 | 0.403214 | 0.792294 | 0.645842 | 0.708806 |
| TCGA-CC-A3MC | 0.773524 | 0.537809 | 0.532846 | 0.869836 | 0.706251 | 0.658832 |
| TCGA-DD-A73A | 0.747025 | 0.289119 | 0.374568 | 0.804144 | 0.619334 | 0.59149 |
| TCGA-DD-AADG | 0.485036 | 0.402359 | 0.437669 | 0.768115 | 0.624849 | 0.71235 |
| TCGA-FV-A2QR | 0.773385 | 0.465353 | 0.510431 | 0.877519 | 0.586998 | 0.725439 |
| TCGA-G3-AAV7 | 0.764147 | 0.599498 | 0.537118 | 0.839 | 0.64109 | 0.504994 |
| TCGA-2Y-A9H4 | 0.7016 | 0.343384 | 0.364204 | 0.751126 | 0.594652 | 0.674559 |
| TCGA-3K-AAZ8 | 0.688097 | 0.205123 | 0.408034 | 0.764736 | 0.675367 | 0.498885 |
| TCGA-CC-5261 | 0.866101 | 0.608486 | 0.617206 | 0.847927 | 0.748511 | 0.79121 |
| TCGA-ZP-A9D2 | 0.731736 | 0.433258 | 0.515516 | 0.785762 | 0.68732 | 0.58015 |
| TCGA-2Y-A9H5 | 0.812341 | 0.473559 | 0.545482 | 0.8445 | 0.761192 | 0.714673 |
| TCGA-G3-A25T | 0.836647 | 0.568572 | 0.509576 | 0.856663 | 0.817711 | 0.60111 |
| TCGA-DD-AAEA | 0.66303 | 0.294804 | 0.355001 | 0.72645 | 0.506556 | 0.438844 |
| TCGA-RG-A7D4 | 0.635322 | 0.122124 | 0.290909 | 0.706129 | 0.57847 | 0.305229 |
| TCGA-DD-AACD | 0.677499 | 0.504122 | 0.50374 | 0.797107 | 0.617896 | 0.63801 |
| TCGA-DD-AAD8 | 0.777602 | 0.355456 | 0.266093 | 0.829534 | 0.57204 | 0.497749 |
| TCGA-5R-AAAM | 0.759179 | 0.664708 | 0.575043 | 0.870795 | 0.78143 | 0.752072 |
| TCGA-EP-A3JL | 0.78303 | 0.499936 | 0.504383 | 0.815957 | 0.639936 | 0.747839 |
| TCGA-DD-A73G | 0.628444 | 0.37568 | 0.326352 | 0.833096 | 0.616572 | 0.555441 |
| TCGA-G3-AAV4 | 0.595922 | 0.661288 | 0.267608 | 0.706325 | 0.623142 | 0.610665 |
| TCGA-ED-A459 | 0.735133 | 0.394141 | 0.42647 | 0.78649 | 0.608444 | 0.451636 |
| TCGA-DD-A73C | 0.677611 | 0.491766 | 0.41717 | 0.732111 | 0.711953 | 0.821412 |
| TCGA-MR-A520 | 0.553444 | 0.31943 | 0.3083 | 0.72399 | 0.657044 | 0.639359 |
| TCGA-RC-A6M3 | 0.728155 | 0.367114 | 0.40788 | 0.760779 | 0.621879 | 0.337226 |
| TCGA-DD-AACU | 0.80119 | 0.394599 | 0.49528 | 0.779265 | 0.68897 | 0.665126 |
| TCGA-WX-AA47 | 0.556711 | 0.525849 | 0.462215 | 0.819692 | 0.60367 | 0.470079 |
| TCGA-DD-A3A4 | 0.57142 | 0.212227 | 0.33004 | 0.785093 | 0.650805 | 0.777101 |
| TCGA-DD-AADQ | 0.595895 | 0.20002 | 0.413281 | 0.805036 | 0.34748 | 0.533579 |
| TCGA-CC-A8HS | 0.632869 | 0.335888 | 0.56544 | 0.801051 | 0.510687 | 0.590147 |
| TCGA-DD-A1EL | 0.611542 | 0.239486 | 0.348297 | 0.702208 | 0.592822 | 0.416209 |
| TCGA-WQ-AB4B | 0.723556 | 0.48836 | 0.568869 | 0.840387 | 0.57887 | 0.691864 |
| TCGA-DD-A11A | 0.721615 | 0.227874 | 0.391125 | 0.716281 | 0.680973 | 0.627642 |
| TCGA-DD-AAD0 | 0.406747 | 0.157497 | 0.053563 | 0.697103 | 0.344695 | 0.32916 |
| TCGA-DD-A3A6 | 0.581078 | 0.730823 | 0.702225 | 0.919129 | 0.540501 | 0.790182 |
| TCGA-DD-A114 | 0.815733 | 0.561656 | 0.646384 | 0.877706 | 0.666306 | 0.679587 |
| TCGA-5C-AAPD | 0.76654 | 0.485066 | 0.456205 | 0.827771 | 0.520251 | 0.585188 |
| TCGA-HP-A5N0 | 0.845821 | 0.467141 | 0.576805 | 0.891738 | 0.679153 | 0.870102 |
| TCGA-T1-A6J8 | 0.749327 | 0.475797 | 0.45339 | 0.792468 | 0.686027 | 0.597776 |
| TCGA-XR-A8TC | 0.745792 | 0.392657 | 0.386388 | 0.770896 | 0.802775 | 0.646414 |
| TCGA-DD-AACK | 0.668468 | 0.337812 | 0.424523 | 0.787256 | 0.678233 | 0.62741 |
| TCGA-MI-A75C | 0.670835 | 0.228527 | 0.277827 | 0.782203 | 0.34136 | 0.377258 |
| TCGA-DD-AACP | 0.56046 | 0.102205 | 0.235786 | 0.730755 | 0.80423 | 0.398083 |
| TCGA-DD-AACT | 0.772939 | 0.505909 | 0.466561 | 0.816343 | 0.733027 | 0.657446 |
| TCGA-DD-A73E | 0.583123 | 0.381366 | 0.321958 | 0.705489 | 0.732661 | 0.546329 |
| TCGA-BC-4072 | 0.798512 | 0.455967 | 0.543305 | 0.846816 | 0.719321 | 0.534257 |
| TCGA-BC-4073 | 0.761913 | 0.517457 | 0.58811 | 0.821895 | 0.640469 | 0.467067 |
| TCGA-XR-A8TG | 0.835599 | 0.42581 | 0.536646 | 0.814169 | 0.602565 | 0.516097 |
| TCGA-RC-A6M6 | 0.720911 | 0.327431 | 0.272155 | 0.764685 | 0.695926 | 0.404089 |
| TCGA-BC-A69H | 0.740525 | 0.439703 | 0.417272 | 0.787783 | 0.629244 | 0.449056 |
| TCGA-DD-AAC8 | 0.462575 | 0.319752 | 0.356495 | 0.74716 | 0.678735 | 0.611159 |
| TCGA-DD-AADJ | 0.429751 | 0.327577 | 0.250104 | 0.775626 | 0.592687 | 0.654989 |
| TCGA-2Y-A9H9 | 0.619785 | 0.292646 | 0.323335 | 0.773311 | 0.561806 | 0.620145 |
| TCGA-ED-A5KG | 0.828184 | 0.542614 | 0.617543 | 0.863751 | 0.717045 | 0.604276 |
| TCGA-DD-AACO | 0.754363 | 0.381501 | 0.425189 | 0.716725 | 0.133388 | 0.583379 |
| TCGA-G3-A25Z | 0.662912 | 0.431611 | 0.451661 | 0.822152 | 0.679837 | 0.725737 |
| TCGA-DD-A4NV | 0.598072 | 0.541133 | 0.300318 | 0.844798 | 0.716613 | 0.760744 |
| TCGA-DD-A39X | 0.685154 | 0.493236 | 0.312849 | 0.816139 | 0.71202 | 0.697956 |
| TCGA-EP-A2KA | 0.670643 | 0.316329 | 0.420565 | 0.739622 | 0.685852 | 0.357285 |
| TCGA-DD-A1EC | 0.658137 | 0.415068 | 0.483185 | 0.844283 | 0.618027 | 0.615742 |
| TCGA-DD-AAD3 | 0.783299 | 0.575054 | 0.59454 | 0.843129 | 0.726653 | 0.778064 |
| TCGA-DD-AACS | 0.758588 | 0.254697 | 0.394003 | 0.755261 | 0.493626 | 0.580041 |
| TCGA-BD-A3ER | 0.831965 | 0.575705 | 0.591401 | 0.843082 | 0.774139 | 0.764521 |
| TCGA-GJ-A6C0 | 0.59885 | 0.500149 | 0.377236 | 0.828619 | 0.612111 | 0.536606 |
| TCGA-2Y-A9GS | 0.816995 | 0.424931 | 0.475408 | 0.749279 | 0.679202 | 0.594831 |
| TCGA-BC-A8YO | 0.62106 | 0.211783 | 0.382375 | 0.72821 | 0.641616 | 0.383334 |
| TCGA-CC-A9FW | 0.622131 | 0.216025 | 0.428302 | 0.744185 | 0.575764 | 0.28315 |
| TCGA-CC-A5UD | 0.65197 | 0.479558 | 0.529017 | 0.765477 | 0.666146 | 0.568003 |
| TCGA-DD-A119 | 0.794786 | 0.535621 | 0.520383 | 0.805631 | 0.633258 | 0.701522 |
| TCGA-DD-AACB | 0.600483 | 0.437836 | 0.339702 | 0.758931 | 0.646983 | 0.49912 |
| TCGA-FV-A495 | 0.719073 | 0.549002 | 0.436697 | 0.826078 | 0.6174 | 0.69026 |
| TCGA-DD-A1EH | 0.742729 | 0.429638 | 0.625169 | 0.820513 | 0.756049 | 0.708292 |
| TCGA-DD-AAEH | 0.723303 | 0.448806 | 0.526716 | 0.814989 | 0.590544 | 0.72265 |
| TCGA-DD-A4NE | 0.751649 | 0.406865 | 0.38552 | 0.824383 | 0.640979 | 0.610102 |
| TCGA-DD-AADV | 0.736134 | 0.502967 | 0.451776 | 0.77686 | 0.691585 | 0.709384 |
| TCGA-DD-AACX | 0.50835 | 0.193871 | 0.282377 | 0.737392 | 0.615028 | 0.474594 |
| TCGA-DD-AACC | 0.675266 | 0.488985 | 0.457516 | 0.832986 | 0.632264 | 0.636565 |
| TCGA-G3-A5SK | 0.640738 | 0.333256 | 0.486237 | 0.696312 | 0.751921 | 0.575867 |
| TCGA-DD-AACV | 0.698128 | 0.172022 | 0.251036 | 0.630779 | 0.458937 | 0.538113 |
| TCGA-CC-5264 | 0.801646 | 0.287831 | 0.489375 | 0.706575 | 0.521179 | 0.421323 |
| TCGA-G3-A7M7 | 0.673901 | 0.402666 | 0.454011 | 0.805092 | 0.672023 | 0.682866 |
| TCGA-G3-AAUZ | 0.725806 | 0.438308 | 0.444925 | 0.78901 | 0.628191 | 0.675216 |
| TCGA-RC-A7SK | 0.779914 | 0.248296 | 0.322868 | 0.711974 | 0.663583 | 0.673518 |
| TCGA-DD-AACI | 0.799702 | 0.399086 | 0.48548 | 0.834945 | 0.626962 | 0.62614 |
| TCGA-2V-A95S | 0.689428 | 0.340059 | 0.476687 | 0.746359 | 0.320175 | 0.546766 |
| TCGA-DD-A4NH | 0.625418 | 0.57102 | 0.524498 | 0.883453 | 0.674707 | 0.460655 |
| TCGA-WX-AA46 | 0.674066 | 0.355627 | 0.516071 | 0.795157 | 0.775554 | 0.68501 |
| TCGA-2Y-A9H2 | 0.80094 | 0.417218 | 0.469778 | 0.74721 | 0.786241 | 0.45355 |
| TCGA-DD-AAW3 | 0.703618 | 0.196824 | 0.423591 | 0.812726 | 0.736021 | 0.67383 |
| TCGA-ED-A82E | 0.849864 | 0.60557 | 0.543064 | 0.807215 | 0.834806 | 0.640003 |
| TCGA-DD-A4NP | 0.546852 | 0.432831 | 0.451127 | 0.737193 | 0.645732 | 0.680231 |
| TCGA-G3-A6UC | 0.668224 | 0.002715 | 0.326859 | 0.720391 | 0.65737 | 0.586689 |
| TCGA-FV-A2QQ | 0.745514 | 0.499094 | 0.454141 | 0.75549 | 0.708215 | 0.682627 |
| TCGA-ZP-A9D4 | 0.559114 | 0.233833 | 0.279573 | 0.703798 | 0.652442 | 0.608038 |
| TCGA-DD-AAE6 | 0.678662 | 0.128235 | 0.274406 | 0.659448 | 0.532473 | 0.417783 |
| TCGA-DD-AACG | 0.598077 | 0.25065 | 0.34007 | 0.725855 | 0.604825 | 0.421037 |
| TCGA-G3-A5SI | 0.676961 | 0.244545 | 0.378153 | 0.752998 | 0.596914 | 0.578515 |
| TCGA-ED-A7PY | 0.712958 | 0.240883 | 0.436986 | 0.767989 | 0.645987 | 0.55995 |
| TCGA-EP-A2KC | 0.672425 | 0.339685 | 0.451401 | 0.775097 | 0.707662 | 0.577005 |
| TCGA-BC-A10X | 0.778155 | 0.58301 | 0.650542 | 0.860746 | 0.861104 | 0.706996 |
| TCGA-CC-A7IH | 0.599836 | 0.104838 | 0.30139 | 0.745557 | 0.690031 | 0.675692 |
| TCGA-DD-A3A8 | 0.522091 | 0.430779 | 0.370357 | 0.732453 | 0.601828 | 0.61348 |
| TCGA-DD-AACL | 0.444772 | 0.422454 | 0.300702 | 0.712292 | 0.46523 | 0.461472 |
| TCGA-DD-A116 | 0.540256 | 0.586553 | 0.387956 | 0.75825 | 0.625843 | 0.641656 |
| TCGA-CC-A7IG | 0.656631 | 0.488408 | 0.351201 | 0.80455 | 0.584013 | 0.521733 |
| TCGA-DD-AAE9 | 0.51838 | 0.286644 | 0.280454 | 0.664028 | 0.586141 | 0.411863 |
| TCGA-DD-AAVP | 0.601811 | 0.509842 | 0.400475 | 0.801611 | 0.533499 | 0.657291 |
| TCGA-G3-AAV5 | 0.645691 | 0.260102 | 0.389466 | 0.745266 | 0.537943 | 0.506244 |
| TCGA-G3-A3CG | 0.525467 | 0.480777 | 0.515511 | 0.831964 | 0.711773 | 0.653691 |
| TCGA-CC-5260 | 0.887995 | 0.625153 | 0.666521 | 0.84342 | 0.86311 | 0.656754 |
| TCGA-CC-A7IJ | 0.526586 | 0.858966 | 0.754356 | 0.922198 | 0.920091 | 0.618249 |
| TCGA-DD-A4NK | 0.608053 | 0.347581 | 0.407237 | 0.820008 | 0.673152 | 0.680674 |
| TCGA-DD-A3A5 | 0.666729 | 0.422004 | 0.436969 | 0.788965 | 0.646195 | 0.609296 |
| TCGA-NI-A4U2 | 0.551222 | 0.353023 | 0.450522 | 0.725387 | 0.637274 | 0.752611 |
| TCGA-DD-A4NF | 0.433653 | 0.29347 | 0.391073 | 0.66406 | 0.735118 | 0.646077 |
| TCGA-DD-AADI | 0.657464 | 0.492756 | 0.574529 | 0.8214 | 0.674563 | 0.629294 |
| TCGA-DD-A4NA | 0.67678 | 0.578177 | 0.583904 | 0.887036 | 0.783734 | 0.414354 |
| TCGA-BC-A10T | 0.756052 | 0.555967 | 0.56913 | 0.85553 | 0.701766 | 0.800982 |
| TCGA-RC-A7SH | 0.781429 | 0.335858 | 0.446274 | 0.807384 | 0.4577 | 0.57302 |
| TCGA-WJ-A86L | 0.548052 | 0.161237 | 0.144397 | 0.653156 | 0.508195 | 0.294168 |
| TCGA-DD-AA3A | 0.640811 | 0.277142 | 0.399636 | 0.755339 | 0.804185 | 0.421894 |
| TCGA-CC-A7IE | 0.756643 | 0.410519 | 0.547909 | 0.80331 | 0.707643 | 0.60851 |
| TCGA-DD-AAVS | 0.805871 | 0.408827 | 0.526565 | 0.822646 | 0.682342 | 0.635237 |
| TCGA-DD-AAE7 | 0.754572 | 0.464727 | 0.514295 | 0.839533 | 0.708768 | 0.743383 |
| TCGA-BC-A3KG | 0.499672 | 0.367175 | 0.092671 | 0.728373 | 0.568531 | 0.406044 |
| TCGA-DD-A3A2 | 0.573489 | 0.389031 | 0.425288 | 0.721597 | 0.68503 | 0.61527 |
| TCGA-DD-AAVR | 0.686536 | 0.606119 | 0.548004 | 0.844538 | 0.762292 | 0.834309 |
| TCGA-CC-A7II | 0.649245 | 0.44722 | 0.610788 | 0.764099 | 0.777168 | 0.505675 |
| TCGA-DD-AACN | 0.751469 | 0.547479 | 0.610786 | 0.827677 | 0.80261 | 0.756682 |
| TCGA-2Y-A9GX | 0.832113 | 0.673337 | 0.631263 | 0.899457 | 0.757684 | 0.742407 |
| TCGA-CC-A3MA | 0.89134 | 0.51774 | 0.67902 | 0.919316 | 0.787662 | 0.675257 |
| TCGA-DD-AACQ | 0.72435 | 0.107984 | 0.38138 | 0.735341 | 0.569498 | 0.541274 |
| TCGA-RC-A7SF | 0.65397 | 0.366951 | 0.417642 | 0.814914 | 0.578327 | 0.525933 |
| TCGA-DD-AADW | 0.706968 | 0.379923 | 0.540462 | 0.870756 | 0.736322 | 0.757928 |
| TCGA-G3-A3CH | 0.659532 | 0.386579 | 0.500259 | 0.834517 | 0.660244 | 0.753411 |
| TCGA-DD-A1EI | 0.678689 | 0.585824 | 0.587056 | 0.832802 | 0.732847 | 0.776929 |
| TCGA-ZP-A9CZ | 0.740658 | 0.358178 | 0.308838 | 0.800074 | 0.552109 | 0.622335 |
| TCGA-KR-A7K0 | 0.701791 | 0.562247 | 0.477564 | 0.830603 | 0.727562 | 0.650102 |
| TCGA-BC-A10Y | 0.661805 | 0.311165 | 0.38146 | 0.77215 | 0.610153 | 0.576066 |
| TCGA-ED-A8O5 | 0.615479 | 0.340711 | 0.535925 | 0.785631 | 0.783213 | 0.757887 |
| TCGA-CC-A9FS | 0.618106 | 0.313152 | 0.399423 | 0.749352 | 0.702653 | 0.6595 |
| TCGA-UB-A7MB | 0.543872 | 0.183525 | 0.236171 | 0.68727 | 0.643672 | 0.547948 |
| TCGA-ED-A627 | 0.857608 | 0.636041 | 0.682184 | 0.908048 | 0.837026 | 0.705441 |
| TCGA-XR-A8TE | 0.594141 | 0.537529 | 0.54115 | 0.768465 | 0.765271 | 0.629497 |
| TCGA-DD-AACF | 0.522424 | 0.234128 | 0.214839 | 0.764786 | 0.561602 | 0.617947 |
| TCGA-K7-A5RF | 0.799947 | 0.502887 | 0.620791 | 0.831771 | 0.789342 | 0.714824 |
| TCGA-ZP-A9D1 | 0.762037 | 0.450424 | 0.551618 | 0.870065 | 0.740555 | 0.768524 |
| TCGA-DD-A3A7 | 0.681176 | 0.373175 | 0.370815 | 0.751304 | 0.466858 | 0.464708 |
| TCGA-DD-AACE | 0.602173 | 0.396413 | 0.330691 | 0.820527 | 0.66944 | 0.713987 |
| TCGA-2Y-A9H3 | 0.70055 | 0.359993 | 0.44382 | 0.839849 | 0.526589 | 0.725505 |
| TCGA-DD-A4NB | 0.705231 | 0.694428 | 0.67345 | 0.919964 | 0.77807 | 0.781486 |
| TCGA-EP-A26S | 0.60692 | 0.335399 | 0.480766 | 0.789181 | 0.599281 | 0.600946 |
| TCGA-DD-AAVX | 0.708059 | 0.291676 | 0.507201 | 0.808894 | 0.674098 | 0.70309 |
| TCGA-ED-A7PZ | 0.729584 | 0.266272 | 0.379841 | 0.831423 | 0.543891 | 0.414347 |
| TCGA-LG-A6GG | 0.631396 | 0.15973 | 0.357264 | 0.769119 | 0.625181 | 0.533994 |
| TCGA-UB-A7ME | 0.815734 | 0.382088 | 0.53311 | 0.808184 | 0.588864 | 0.684733 |
| TCGA-G3-A7M9 | 0.703024 | 0.469355 | 0.523488 | 0.714527 | 0.665616 | 0.354557 |
| TCGA-DD-AACJ | 0.568233 | 0.201512 | 0.204789 | 0.718056 | 0.599134 | 0.532122 |
| TCGA-LG-A9QD | 0.674657 | 0.388649 | 0.461853 | 0.844485 | 0.649475 | 0.653881 |
| TCGA-ZS-A9CG | 0.519179 | 0.48177 | 0.394563 | 0.780578 | 0.635372 | 0.670402 |
| TCGA-K7-A6G5 | 0.693018 | 0.488746 | 0.472011 | 0.778701 | 0.672656 | 0.64091 |
| TCGA-DD-AADS | 0.667418 | 0.249031 | 0.375974 | 0.729478 | 0.179676 | 0.605204 |
| TCGA-DD-AAW1 | 0.45227 | 0.313883 | 0.422109 | 0.79797 | 0.64366 | 0.636128 |
| TCGA-DD-A118 | 0.660941 | 0.482206 | 0.535742 | 0.817583 | 0.730267 | 0.714467 |
| TCGA-DD-AADA | 0.643618 | 0.368111 | 0.460187 | 0.831505 | 0.680121 | 0.783015 |
| TCGA-G3-AAV1 | 0.689368 | 0.379968 | 0.480482 | 0.79547 | 0.718569 | 0.701319 |
| TCGA-O8-A75V | 0.770978 | 0.535928 | 0.486381 | 0.815112 | 0.677877 | 0.696442 |
| TCGA-ZP-A9CY | 0.716405 | 0.486218 | 0.481917 | 0.819185 | 0.632026 | 0.734841 |
| TCGA-G3-A7M8 | 0.682062 | 0.435248 | 0.375438 | 0.776178 | 0.67085 | 0.721798 |
| TCGA-DD-AAE0 | 0.805678 | 0.50004 | 0.588436 | 0.867584 | 0.686136 | 0.529696 |

**Supplementary Table S3.** Detailed data of different stromal-activation relevant signatures based on GEO dataset (GSE14520).

| id | EMT1 | EMT2 | EMT3 | Pan-F-TBRS | Wnt target |
| --- | --- | --- | --- | --- | --- |
| GSM362958 | 0.562578 | 0.411136 | 0.462367 | 0.600284 | 0.614264 |
| GSM362959 | 0.49258 | 0.38107 | 0.344666 | 0.773515 | 0.837437 |
| GSM362960 | 0.552457 | 0.377294 | 0.581681 | 0.747432 | 0.862167 |
| GSM362964 | 0.648771 | 0.382379 | 0.466024 | 0.725785 | 0.849959 |
| GSM362965 | 0.55903 | 0.586914 | 0.357967 | 0.68561 | 0.759593 |
| GSM362966 | 0.750713 | 0.329129 | 0.443478 | 0.660767 | 0.81535 |
| GSM362970 | 0.479952 | 0.558619 | 0.498063 | 0.736023 | 0.802448 |
| GSM362971 | 0.721503 | 0.455614 | 0.651131 | 0.784295 | 0.768232 |
| GSM362972 | 0.549638 | 0.408765 | 0.175055 | 0.68426 | 0.773209 |
| GSM362976 | 0.708899 | 0.416036 | 0.481776 | 0.724049 | 0.823644 |
| GSM362977 | 0.717497 | 0.304658 | 0.357706 | 0.61857 | 0.620464 |
| GSM362978 | 0.729466 | 0.329474 | 0.242194 | 0.678918 | 0.660464 |
| GSM362982 | 0.67632 | 0.424644 | 0.298508 | 0.69052 | 0.824958 |
| GSM362983 | 0.533099 | 0.263163 | 0.40026 | 0.512399 | 0.706762 |
| GSM362984 | 0.477902 | 0.323708 | 0.419568 | 0.633684 | 0.855928 |
| GSM362986 | 0.528858 | 0.383794 | 0.275652 | 0.671501 | 0.834306 |
| GSM362987 | 0.500369 | 0.316052 | 0.353308 | 0.591525 | 0.758464 |
| GSM362988 | 0.641323 | 0.286061 | 0.471707 | 0.580857 | 0.693943 |
| GSM362992 | 0.59888 | 0.200538 | 0.365032 | 0.603532 | 0.824829 |
| GSM362993 | 0.874714 | 0.419394 | 0.594079 | 0.700314 | 0.807887 |
| GSM362994 | 0.549752 | 0.403057 | 0.333246 | 0.640158 | 0.663869 |
| GSM363008 | 0.70326 | 0.443164 | 0.685452 | 0.792556 | 0.837367 |
| GSM363009 | 0.585311 | 0.429197 | 0.571591 | 0.744569 | 0.772802 |
| GSM363010 | 0.80503 | 0.469191 | 0.57371 | 0.778067 | 0.918333 |
| GSM363011 | 0.599402 | 0.305325 | 0.324705 | 0.643246 | 0.768559 |
| GSM363012 | 0.718098 | 0.409278 | 0.529051 | 0.688035 | 0.849291 |
| GSM363013 | 0.654809 | 0.457899 | 0.592628 | 0.711925 | 0.834431 |
| GSM363014 | 0.787684 | 0.362711 | 0.57833 | 0.676748 | 0.771361 |
| GSM363015 | 0.754117 | 0.412403 | 0.640572 | 0.745071 | 0.806393 |
| GSM363016 | 0.534172 | 0.301097 | 0.407323 | 0.576602 | 0.84115 |
| GSM363017 | 0.68338 | 0.547145 | 0.464112 | 0.767508 | 0.808063 |
| GSM363029 | 0.649736 | 0.593378 | 0.540179 | 0.76567 | 0.738137 |
| GSM363030 | 0.703576 | 0.298474 | 0.632784 | 0.728431 | 0.750503 |
| GSM363031 | 0.689267 | 0.339047 | 0.489824 | 0.705489 | 0.835172 |
| GSM363032 | 0.589504 | 0.23777 | 0.440709 | 0.585929 | 0.819494 |
| GSM363033 | 0.521356 | 0.168128 | 0.546903 | 0.650916 | 0.710148 |
| GSM363034 | 0.700145 | 0.302461 | 0.471921 | 0.707911 | 0.745285 |
| GSM363035 | 0.760505 | 0.289465 | 0.42666 | 0.748593 | 0.806162 |
| GSM363036 | 0.59424 | 0.241324 | 0.478351 | 0.592763 | 0.832062 |
| GSM363037 | 0.541046 | 0.421924 | 0.488536 | 0.530473 | 0.808727 |
| GSM363038 | 0.622451 | 0.426341 | 0.220022 | 0.663533 | 0.828761 |
| GSM363039 | 0.633916 | 0.402524 | 0.279284 | 0.556389 | 0.795914 |
| GSM363048 | 0.820412 | 0.21321 | 0.546458 | 0.646468 | 0.822061 |
| GSM363049 | 0.632857 | 0.249842 | 0.411686 | 0.605382 | 0.836475 |
| GSM363050 | 0.839529 | 0.345015 | 0.601586 | 0.777356 | 0.866889 |
| GSM363051 | 0.655757 | 0.075675 | 0.499586 | 0.728362 | 0.951151 |
| GSM363052 | 0.621492 | 0.383729 | 0.530654 | 0.665694 | 0.921582 |
| GSM363053 | 0.674931 | 0.312911 | 0.471973 | 0.701591 | 0.769147 |
| GSM363054 | 0.767108 | 0.376033 | 0.791696 | 0.773903 | 0.728514 |
| GSM363055 | 0.880961 | 0.527511 | 0.857779 | 0.850493 | 0.93287 |
| GSM363056 | 0.830038 | 0.342891 | 0.594872 | 0.62298 | 0.563012 |
| GSM363057 | 0.659569 | 0.276356 | 0.389717 | 0.578952 | 0.852333 |
| GSM363069 | 0.61142 | 0.406387 | 0.34959 | 0.687934 | 0.821948 |
| GSM363070 | 0.647005 | 0.292279 | 0.554049 | 0.609373 | 0.884194 |
| GSM363071 | 0.652921 | 0.369981 | 0.322243 | 0.638832 | 0.788394 |
| GSM363072 | 0.573048 | 0.581717 | 0.153825 | 0.573281 | 0.619591 |
| GSM363073 | 0.657784 | 0.252504 | 0.614887 | 0.692664 | 0.894418 |
| GSM363074 | 0.788767 | 0.507666 | 0.633889 | 0.755691 | 0.792036 |
| GSM363075 | 0.588242 | 0.408782 | 0.510634 | 0.703305 | 0.817719 |
| GSM363076 | 0.656751 | 0.360381 | 0.543149 | 0.835442 | 0.790958 |
| GSM363077 | 0.609522 | 0.30254 | 0.24889 | 0.612088 | 0.84912 |
| GSM363078 | 0.420419 | 0.399266 | 0.274206 | 0.654918 | 0.799952 |
| GSM363079 | 0.616313 | 0.465403 | 0.571202 | 0.776064 | 0.81621 |
| GSM363080 | 0.709168 | 0.327797 | 0.398548 | 0.717241 | 0.903155 |
| GSM363081 | 0.566892 | 0.421128 | 0.444569 | 0.666807 | 0.803698 |
| GSM363082 | 0.714756 | 0.199611 | 0.487785 | 0.599303 | 0.739182 |
| GSM363083 | 0.522786 | 0.457581 | 0.738586 | 0.789129 | 0.905482 |
| GSM363084 | 0.596165 | 0.304344 | 0.451488 | 0.693715 | 0.790615 |
| GSM363085 | 0.526949 | 0.220218 | 0.669083 | 0.652185 | 0.850939 |
| GSM363086 | 0.782159 | 0.291218 | 0.451711 | 0.599849 | 0.709541 |
| GSM363087 | 0.67639 | 0.478259 | 0.416706 | 0.741232 | 0.786845 |
| GSM363098 | 0.475464 | 0.201342 | 0.576691 | 0.682897 | 0.839386 |
| GSM363099 | 0.636473 | 0.432666 | 0.595154 | 0.726707 | 0.807903 |
| GSM363100 | 0.75275 | 0.309744 | 0.657798 | 0.655635 | 0.767173 |
| GSM363101 | 0.621617 | 0.316727 | 0.555311 | 0.641101 | 0.858865 |
| GSM363102 | 0.693305 | 0.381347 | 0.563301 | 0.643426 | 0.741134 |
| GSM363104 | 0.573287 | 0.34573 | 0.514756 | 0.62285 | 0.80806 |
| GSM363105 | 0.576014 | 0.441288 | 0.37794 | 0.718288 | 0.820233 |
| GSM363106 | 0.604231 | 0.141346 | 0.362761 | 0.528782 | 0.830757 |
| GSM363107 | 0.548906 | 0.408842 | 0.584036 | 0.645131 | 0.663355 |
| GSM363108 | 0.720561 | 0.335849 | 0.51861 | 0.681767 | 0.848743 |
| GSM363109 | 0.522339 | 0.216139 | 0.56616 | 0.563749 | 0.794214 |
| GSM363115 | 0.705628 | 0.360145 | 0.579352 | 0.683174 | 0.741708 |
| GSM363121 | 0.573377 | 0.40784 | 0.621932 | 0.735527 | 0.909149 |
| GSM363122 | 0.584555 | 0.248888 | 0.459592 | 0.689463 | 0.776524 |
| GSM363123 | 0.574271 | 0.337565 | 0.377675 | 0.492618 | 0.742756 |
| GSM363124 | 0.712995 | 0.488211 | 0.402622 | 0.78254 | 0.804263 |
| GSM363125 | 0.635038 | 0.412683 | 0.598653 | 0.713835 | 0.814672 |
| GSM363126 | 0.597327 | 0.162197 | 0.48422 | 0.632001 | 0.690449 |
| GSM363127 | 0.549267 | 0.300228 | 0.413821 | 0.577893 | 0.809832 |
| GSM363128 | 0.638885 | 0.252068 | 0.510522 | 0.622818 | 0.823264 |
| GSM363129 | 0.623029 | 0.314414 | 0.438455 | 0.610973 | 0.860951 |
| GSM363130 | 0.592794 | 0.112536 | 0.180482 | 0.50156 | 0.859263 |
| GSM363142 | 0.815919 | 0.36522 | 0.405614 | 0.652068 | 0.813385 |
| GSM363143 | 0.609612 | 0.488987 | 0.620715 | 0.782314 | 0.742478 |
| GSM363144 | 0.768566 | 0.439833 | 0.629644 | 0.688305 | 0.829172 |
| GSM363145 | 0.608464 | 0.214494 | 0.605033 | 0.645937 | 0.86721 |
| GSM363146 | 0.587206 | 0.539948 | 0.655311 | 0.726217 | 0.792915 |
| GSM363147 | 0.478962 | 0.296443 | 0.434691 | 0.638717 | 0.882413 |
| GSM363148 | 0.57781 | 0.144176 | 0.036346 | 0.556146 | 0.835827 |
| GSM363149 | 0.754347 | 0.28135 | 0.339888 | 0.594955 | 0.663292 |
| GSM363150 | 0.596881 | 0.31286 | 0.596982 | 0.58121 | 0.898574 |
| GSM363151 | 0.56548 | 0.341141 | 0.060211 | 0.590437 | 0.838692 |
| GSM363152 | 0.88323 | 0.347381 | 0.466049 | 0.717582 | 0.891585 |
| GSM363164 | 1 | 0.378587 | 0.606419 | 0.746932 | 0.841081 |
| GSM363166 | 0.689919 | 0.183333 | 0.184086 | 0.52578 | 0.633463 |
| GSM363168 | 0.852374 | 0.177805 | 0.580929 | 0.599895 | 0.749398 |
| GSM363169 | 0.529429 | 0.405974 | 0.503383 | 0.612211 | 0.852312 |
| GSM363170 | 0.651788 | 0.558967 | 0.374036 | 0.651756 | 0.721277 |
| GSM363172 | 0.573365 | 0.476547 | 0.405338 | 0.694378 | 0.769593 |
| GSM363174 | 0.517659 | 0.31071 | 0.372331 | 0.602965 | 0.872849 |
| GSM363176 | 0.683838 | 0.371937 | 0.528968 | 0.706676 | 0.822975 |
| GSM363178 | 0.645178 | 0.342126 | 0.487335 | 0.647737 | 0.849836 |
| GSM363180 | 0.521315 | 0.290157 | 0.421234 | 0.689314 | 0.848401 |
| GSM363182 | 0.680945 | 0.121522 | 0.357737 | 0.512829 | 0.703083 |
| GSM363184 | 0.834701 | 0.654373 | 0.686167 | 0.698512 | 0.854938 |
| GSM363186 | 0.655636 | 0.458477 | 0.562772 | 0.748071 | 0.799817 |
| GSM363188 | 0.656536 | 0.523176 | 0.577085 | 0.735754 | 0.812021 |
| GSM363190 | 0.618522 | 0.538486 | 0.526038 | 0.706938 | 0.816647 |
| GSM363192 | 0.650493 | 0.214383 | 0.350816 | 0.562994 | 0.797245 |
| GSM363194 | 0.553794 | 0.339214 | 0.386412 | 0.591406 | 0.848444 |
| GSM363196 | 0.721623 | 0.417141 | 0.315704 | 0.591834 | 0.741467 |
| GSM363198 | 0.535319 | 0.295531 | 0.444963 | 0.695437 | 0.874346 |
| GSM363200 | 0.59919 | 0.453897 | 0.607716 | 0.73291 | 0.804206 |
| GSM363202 | 0.608235 | 0.364607 | 0.167149 | 0.576766 | 0.751587 |
| GSM363204 | 0.82952 | 0.379594 | 0.615869 | 0.716233 | 0.87622 |
| GSM363205 | 0.666425 | 0.284061 | 0.499395 | 0.616332 | 0.740612 |
| GSM363207 | 0.614998 | 0.492867 | 0.438077 | 0.666764 | 0.845354 |
| GSM363209 | 0.699193 | 0.410288 | 0.430027 | 0.684478 | 0.813487 |
| GSM363211 | 0.590659 | 0.305004 | 0.617439 | 0.701653 | 0.828974 |
| GSM363213 | 0.767025 | 0.292051 | 0.459915 | 0.621168 | 0.873339 |
| GSM363215 | 0.569158 | 0.417823 | 0.566205 | 0.699185 | 0.843211 |
| GSM363217 | 0.714511 | 0.250185 | 0.127849 | 0.538223 | 0.731139 |
| GSM363218 | 0.767857 | 0.259277 | 0.130554 | 0.628873 | 0.878569 |
| GSM363220 | 0.529679 | 0.338602 | 0.426075 | 0.673375 | 0.734831 |
| GSM363222 | 0.738369 | 0.352024 | 0.534636 | 0.644845 | 0.86852 |
| GSM363224 | 0.657235 | 0.407628 | 0.354945 | 0.612966 | 0.64671 |
| GSM363226 | 0.664802 | 0.355256 | 0.452522 | 0.630196 | 0.838791 |
| GSM363228 | 0.702974 | 0.208856 | 0.492998 | 0.591969 | 0.722129 |
| GSM363230 | 0.655082 | 0.162572 | 0.40637 | 0.585762 | 0.713347 |
| GSM363232 | 0.693629 | 0.123601 | 0.521826 | 0.480658 | 0.793462 |
| GSM363235 | 0.538625 | 0.409855 | 0.412281 | 0.679622 | 0.830993 |
| GSM363237 | 0.421796 | 0.312509 | 0.460495 | 0.595895 | 0.797343 |
| GSM363239 | 0.582019 | 0.433999 | 0.543141 | 0.675953 | 0.73266 |
| GSM363241 | 0.717217 | 0.305272 | 0.459163 | 0.578034 | 0.614703 |
| GSM363243 | 0.683974 | 0.454376 | 0.51205 | 0.703728 | 0.866908 |
| GSM363245 | 0.486414 | 0.308965 | 0.325748 | 0.640092 | 0.789234 |
| GSM363247 | 0.76996 | 0.451662 | 0.436671 | 0.620383 | 0.804464 |
| GSM363249 | 0.61134 | 0.294958 | 0.639273 | 0.57509 | 0.925618 |
| GSM363251 | 0.627363 | 0.449931 | 0.394145 | 0.703339 | 0.849694 |
| GSM363263 | 0.587143 | 0.389013 | 0.516148 | 0.616704 | 0.813582 |
| GSM363264 | 0.673481 | 0.271505 | 0.640527 | 0.596178 | 0.826867 |
| GSM363265 | 0.619496 | 0.390284 | 0.624771 | 0.775085 | 0.856771 |
| GSM363266 | 0.553178 | 0.229782 | 0.352163 | 0.631654 | 0.782071 |
| GSM363267 | 0.654787 | 0.333254 | 0.544722 | 0.672516 | 0.7738 |
| GSM363268 | 0.573128 | 0.283067 | 0.355552 | 0.59862 | 0.87119 |
| GSM363269 | 0.742538 | 0.169071 | 0.158641 | 0.594255 | 0.654058 |
| GSM363270 | 0.699846 | 0.296919 | 0.361119 | 0.675721 | 0.803229 |
| GSM363271 | 0.696407 | 0.641336 | 0.693045 | 0.811923 | 0.852679 |
| GSM363272 | 0.729779 | 0.362655 | 0.449014 | 0.683104 | 0.820107 |
| GSM363273 | 0.717705 | 0.324846 | 0.445257 | 0.654891 | 0.673136 |
| GSM363274 | 0.621835 | 0.217374 | 0.437554 | 0.592067 | 0.800804 |
| GSM363275 | 0.659559 | 0.37001 | 0.495248 | 0.702318 | 0.835098 |
| GSM363288 | 0.622521 | 0.435062 | 0.527111 | 0.668102 | 0.739129 |
| GSM363289 | 0.954805 | 0.461123 | 0.632872 | 0.705425 | 0.669337 |
| GSM363290 | 0.521624 | 0.259177 | 0.552355 | 0.689846 | 0.709142 |
| GSM363291 | 0.603323 | 0.269484 | 0.252963 | 0.624524 | 0.747192 |
| GSM363292 | 0.585875 | 0.258783 | 0.559634 | 0.606381 | 0.822023 |
| GSM363293 | 0.59203 | 0.421445 | 0.589898 | 0.671639 | 0.842069 |
| GSM363294 | 0.598707 | 0.583875 | 0.587841 | 0.814486 | 0.833919 |
| GSM363295 | 0.624513 | 0.286593 | 0.377726 | 0.663286 | 0.753423 |
| GSM363296 | 0.521851 | 0.350121 | 0.404995 | 0.703768 | 0.858744 |
| GSM363297 | 0.471479 | 0.37404 | 0.513561 | 0.63924 | 0.871749 |
| GSM363298 | 0.855403 | 0.398512 | 0.671334 | 0.745173 | 0.799237 |
| GSM363309 | 0.557553 | 0.283732 | 0.325456 | 0.681311 | 0.84926 |
| GSM363310 | 0.63147 | 0.421895 | 0.326373 | 0.74161 | 0.828976 |
| GSM363311 | 0.710501 | 0.301643 | 0.424512 | 0.645588 | 0.739876 |
| GSM363312 | 0.656695 | 0.334183 | 0.467921 | 0.634494 | 0.662056 |
| GSM363313 | 0.784089 | 0.25537 | 0.445076 | 0.640668 | 0.830614 |
| GSM363314 | 0.579667 | 0.283227 | 0.024575 | 0.613367 | 0.743558 |
| GSM363315 | 0.89792 | 0.342974 | 0.589549 | 0.644316 | 0.757634 |
| GSM363316 | 0.946886 | 0.268605 | 0.702811 | 0.699982 | 0.695039 |
| GSM363317 | 0.533668 | 0.341491 | 0.543315 | 0.63488 | 0.855946 |
| GSM363326 | 0.581119 | 0.331056 | 0.534157 | 0.673057 | 0.818811 |
| GSM363327 | 0.758106 | 0.452288 | 0.549946 | 0.694997 | 0.738684 |
| GSM363328 | 0.653728 | 0.414613 | 0.487657 | 0.570522 | 0.818379 |
| GSM363329 | 0.774688 | 0.376365 | 0.419651 | 0.633875 | 0.827712 |
| GSM363330 | 0.583006 | 0.264664 | 0.486536 | 0.645839 | 0.912824 |
| GSM363331 | 0.761342 | 0.15908 | 0.350359 | 0.559308 | 0.820244 |
| GSM363332 | 0.911257 | 0.279475 | 0.654248 | 0.648499 | 0.849014 |
| GSM363333 | 0.696008 | 0.576785 | 0.642518 | 0.840772 | 0.854023 |
| GSM363334 | 0.749795 | 0.444533 | 0.552092 | 0.694429 | 0.791447 |
| GSM363335 | 0.614822 | 0.420469 | 0.487128 | 0.711666 | 0.842105 |
| GSM363336 | 0.546285 | 0.408558 | 0.481895 | 0.676913 | 0.850525 |
| GSM363337 | 0.766048 | 0.419882 | 0.657771 | 0.754145 | 0.843247 |
| GSM363339 | 0.594358 | 0.402507 | 0.440394 | 0.705864 | 0.816448 |
| GSM363341 | 0.5359 | 0.154697 | 0.316124 | 0.613243 | 0.837777 |
| GSM363343 | 0.502764 | 0.363514 | 0.435006 | 0.770341 | 0.862602 |
| GSM363344 | 0.590387 | 0.396447 | 0.495761 | 0.595473 | 0.707035 |
| GSM363346 | 0.630793 | 0.262431 | 0.522406 | 0.587903 | 0.687841 |
| GSM363348 | 0.714517 | 0.281956 | 0.67076 | 0.695598 | 0.889875 |
| GSM363350 | 0.565581 | 0.484604 | 0.376111 | 0.710957 | 0.817629 |
| GSM363352 | 0.636619 | 0.50321 | 0.572088 | 0.766239 | 0.801877 |
| GSM363354 | 0.683392 | 0.390482 | 0.357561 | 0.558345 | 0.728678 |
| GSM363355 | 0.706351 | 0.438711 | 0.394673 | 0.693744 | 0.817857 |
| GSM363357 | 0.484506 | 0.228781 | 0.493365 | 0.630784 | 0.85614 |
| GSM363358 | 0.521852 | 0.314848 | 0.453734 | 0.587199 | 0.732651 |
| GSM363360 | 0.894089 | 0.386575 | 0.643778 | 0.698899 | 0.795968 |
| GSM363362 | 0.511863 | 0.351416 | 0.478035 | 0.653414 | 0.824245 |
| GSM363364 | 0.486788 | 0.29533 | 0.482918 | 0.634397 | 0.834003 |
| GSM363366 | 0.668672 | 0.375313 | 0.304802 | 0.563411 | 0.647256 |
| GSM363368 | 0.653137 | 0.349476 | 0.440865 | 0.595997 | 0.787981 |
| GSM363371 | 0.539849 | 0.205998 | 0.558695 | 0.559502 | 0.740046 |
| GSM363376 | 0.49175 | 0.492048 | 0.497801 | 0.742813 | 0.846566 |
| GSM363378 | 0.693587 | 0.289137 | 0.555598 | 0.627644 | 0.817896 |
| GSM363384 | 0.51961 | 0.40084 | 0.310084 | 0.600059 | 0.833139 |
| GSM363386 | 0.52114 | 0.549027 | 0.539417 | 0.725406 | 0.773683 |
| GSM363388 | 0.450314 | 0.536814 | 0.457993 | 0.704455 | 0.770835 |
| GSM363391 | 0.640382 | 0.289526 | 0.553218 | 0.68082 | 0.816171 |
| GSM363393 | 0.445269 | 0.610798 | 0.662883 | 0.88956 | 0.769874 |
| GSM363400 | 0.609881 | 0.300611 | 0.112183 | 0.55742 | 0.627208 |
| GSM363404 | 0.502227 | 0.296627 | 0 | 0.662718 | 0.797843 |
| GSM712532 | 0.555908 | 0.310481 | 0.403178 | 0.593706 | 0.838831 |
| GSM712534 | 0.566741 | 0.369133 | 0.458082 | 0.703189 | 0.804551 |
| GSM712542 | 0.642376 | 0.174181 | 0.621416 | 0.596037 | 0.874655 |
