# Supplementary material for: 27‐Hydroxycholesterol promotes metastasis by SULT2A1‐dependent alteration in hepatocellular carcinoma
Source: Cancer Sci. 2022 Jun 13;113(8):2575–89. doi: 10.1111/cas.15435 (PMC9357618; doi:10.1111/cas.15435)
Supplement: Supplementary file 3 — Appendix S1 [file CAS-113-2575-s002.docx]

Appendix S1

**27-Hydroxycholesterol Promotes Metastasis by SULT2A1-Dependent Alteration in Hepatocellular Carcinoma**

Taochen He^1,2, *^, Baorui Tao^1,2, *^, Chenhe Yi^1,2, *^, Chong Zhang^1,2^, Peng Zhang^1,2^, Weiqing Shao^1^, Yitong Li^1,2^, Zhenmei Chen^1,2^, Lu Lu^1^, Huliang Jia^1^, Wenwei Zhu^1^, Jing Lin^1^, Jinhong Chen^1,2^

*These authors contributed equally to this work.

Authors’ affiliations: ^1^Department of General Surgery, Huashan Hospital, Fudan University, Shanghai, China

^2^Institute of Cancer Metastasis, Fudan University, Shanghai, China

Corresponding author: Jinhong Chen and Jing Lin, Department of General Surgery, Huashan Hospital, Fudan University. 12 Wulumuqi Road (M), Shanghai 200040, China. Tel: +86-21-52887170. E-mail: [jinhongch@hotmail.com](mailto:jinhongch@hotmail.com); Linjingfdu@163.com

**Material and Methods**

**Reagents**

25-OHC (HY-113134), 27-OHC (HY-N2371) and Bay11-7082 (HY-13453) were purchased from MedChemExpress, China. E-cadherin (20874-1-AP), N-cadherin (22018-1-AP), GAPDH (60004-1-lg) were purchased from Proteintech; SULT2A1 (ab194113) was purchased from Abcam; p65 (T55034) and p-p65 (TP56372) were purchased from Abmart; Twist1 (GTX50821), slug (GTX128796), snail (GTX125918) was purchased from GeneTex.

**Cell Culture**

All cell lines were obtained from the Institute of cancer metastasis, Fudan University, Shanghai, China. Cells were maintained in DMEM (GIBCO) supplemented with 10% FBS (GIBCO) and 1% penicillin and streptomycin (Beyotime Biotech Co. Ltd., China) at 37℃ in 5% CO_2_. For oxysterols and Bay11-7082 supplement, cells were pretreated with required concentrations of reagents for 72h.

**Animal Studies**

6 weeks old male nude mice (BALB/c nu/nu) were obtained from Shanghai Jihui Laboratory Animal Care Co.,Ltd (Shanghai, China) and were randomly allocated to each group. All the experiments were performed based on the protocols approved by the institutional review board of Department of Laboratory Animal Science, Fudan University, and conformed to the National Guidelines for Animal Usage in Research.

**Animal Models**

For subcutaneous xenograft models, Huh7 (together with shSULT2A1 cells) or HCC-LM3 cells (together with overexpression of SULT2A1 cells) were pre-treated with or without 2μM 27-OHC for two weeks and then 2*10^6^ cells were injected subcutaneously into six mice per group to establish the models. Specially, 20mg/kg 27-OHC or placebo was injected intratumorally twice a week. Tumor volume were measured every three days by a caliper and calculated based on the formula: volume=(Length*Width^2^)/2. Mice were sacrificed after 28 days and tumors were removed and taken photos.

For orthotopic xenograft models, subcutaneous tumors from each subcutaneous xenograft group were removed and dissected into 1mm^3^ sections and then incubated into livers of six mice per group. Specially, for SULT2A1 overexpression models, 20mg/kg 27-OHC or placebo was injected subcutaneously per day; for shSULT2A1 models, 20mg/kg 27-OHC was injected subcutaneously per day; 10mg/kg Bay11-7082 was injected intraperitoneally three times per week. Mice were sacrificed after 6 weeks and tumors livers and lungs were removed, fixed in 10% formalin and embedded in paraffin. Consecutive sections were made for every lung tissue and HE staining was performed according to routine protocols. For each lung, the average metastatic lesions of 3 evenly spaced sections were quantified and averaged to evaluate the number of lung metastasis.

**Clinical Specimens**

All HCC tissues between 2018 and 2020 were obtained from Department of General Surgery, Huashan hospital, Fudan university (Shanghai, China). Clinical samples were collected from patients after informed consent in accordance with a protocol approved by the Ethics Committee of Huashan hospital, Fudan University (Shanghai, China). The microvascular invasion (MVI) grade was confirmed by two independent pathologists from Department of Pathology, Huashan hospital, Fudan university (Shanghai, China).

**Lentivirus-Mediated Regulation of Gene Expression**

Lentivirus generation was performed as described previously.^1^ Briefly, SULT2A1 lentiviral overexpression vector was obtained by cloning human SULT2A1 coding sequence into pCDH vector. shRNA of SULT2A1 were obtained into pLKO vector by the primer sequences listed as follows: shSULT2A1 1# F, 5’- CCGGACGGAGAGTCCACGTTTATTCCTCGAGGAATAAACGTGGACTCTCCGTTTTTTTG-3’ and R, 5’- AATTCAAAAAAACGGAGAGTCCACGTTTATTCCTCGAGGAATAAACGTGGACTCTCCGT-3’; shSULT2A1 2# F, 5’- CCGGGTACGTGATGAGTTCGTGATACTCGAGTATCACGAACTCATCACGTACTTTTTG-3’ and R, 5’- AATTCAAAAAGTACGTGATGAGTTCGTGATACTCGAGTATCACGAACTCATCACGTAC-3’.

**Tumor Microarray (TMA)**

TMA was obtained from the previous research.^2^ Briefly, paired primary HCC tissues (HT), adjacent non-tumor liver tissues (NLT) and portal vein tumor thrombus tissues (PVTT) from 37 patients were obtained from Department of General Surgery, Huashan hospital, Fudan university (Shanghai, China). IHC score was computed as: 0, negative; 1, weak; 2, moderate; 3, strong.

**Immunohistochemistry**

Liver sections from the orthotopic xenograft models were first deparaffinized and rehydrated and then blocked with 3% H_2_O_2_ for 10 min. Then the following processes were performed as normal. The staining extent score was on a scale of 0 to 3, corresponding to the percentage of immunoreactive cells and the staining intensity. IHC H-score was computed as the sum of 3*(% 3) + 2*(% 2) + 1*(% 1) + 0*(% 0). (0, negative; 1, weak; 2, moderate; 3, strong)

**Oxysterol Measurements**

For ***in vitro*** experiments, the intracellular oxysterols of HCC cell lines were measured. Cells were digested with trypsin, washed with cold PBS and then sonicated. Supernatant was recovered and used to measure the level of oxysterol. For ***in vivo*** experiments, the oxysterol concentrations of tumor tissues were measured according to manufacturer’s instructions. 25-OHC was determined using 25-OHC ELISA Kit (ml061349) and 27-OHC was determined using 27-OHC ELISA Kit (ml063097) according to the manufacturer’s instructions. Both ELISA Kits were purchased from Shanghai Enzyme-linked Biotechnology Co., Ltd.

**Quantitive RT-PCR**

Quantitive RT-PCR was performed as described previously.^1^ Primer sets for quantitative RT-PCR were listed as follows: SULT2A1 F, 5’- CGTGATGAGTTCGTGATAAGGG-3’ and R, 5’- GGCAGAGAATCTCAGCCAACC-3’; ICAM1 F, 5’- ATGCCCAGACATCTGTGTCC-3’ and R, 5’- GGGGTCTCTATGCCCAACAA-3’; VCAM1 F, 5’- GGGAAGATGGTCGTGATCCTT-3’ and R, 5’- TCTGGGGTGGTCTCGATTTTA-3’; MMP9 F, 5’- GGGACGCAGACATCGTCATC-3’ and R, 5’- TCGTCATCGTCGAAATGGGC-3’; GAPDH F, 5’- ACAACTTTGGTATCGTGGAAGG-3’ and R, 5’- GCCATCACGCCACAGTTTC-3’.

**Cell Migration Assay**

The wound scratch assay was used to assess cell migration. Cells for each group were seeded and cultured in 6-well plates until confluent cell monolayers had been formed. The adherent monolayer cells were scratched using 200μl pipette tips and were then cultured with DMEM containing 1% FBS after washed 3 times with PBS. 5 Images of the scratch wounds per well were captured at the indicated times and the coordinate for each image was documented based on the Leica microsystem (Leica Application Suite, version 4.4.0) to minimize the variance. Percentage of wound closure was calculated as the following formula: (original scratch width—scratch width after healing) * (original scratch width) ^−1^ * 100%.

**Cell Invasion Assay**

The cell invasion assay was performed as described previously.^1^ Briefly, different cells treated as indicated previously were seeded at 5*10^4^ in serum-free medium with or without respective oxysterols in the top chamber and were left to migrate for 72h at 37℃ in 5% CO_2_. Then the filters were stained with Giemsa, imaged, and counted with the Leica microsystem (Leica Application Suite, version 4.4.0). The cell numbers from 5 representative photos of each group were used for statistical analysis.

**Bioinformatics analysis**

We downloaded four hepatocellular carcinoma datasets for subsequent analysis. RNA-seq expression profiles (TCGA-LIHC, FPKM value) and relevant clinical information were acquired from The Cancer Genome Atlas database (TCGA, https://portal.gdc.cancer.gov/). Microarray data (GSE9843, GSE76427, GSE14520) and related clinical information were obtained from the Gene Expression Omnibus database (GEO, https://www.ncbi.nlm.nih.gov/geo/). Survminer and survival R packages were utilized to perform survival analysis and select the optimal cutoff value. Single sample gene set enrichment analysis (ssGSEA) algorithm was implemented to evaluate the stromal-activated status of each patient. Relevant geneset signatures were collected from previous published literature, including EMT1, EMT2, EMT3, Pan-F-TBRS, Wnt target and Angiogenesis. R package GSVA was employed for calculating enrichment scores. Additionally, we used the pROC package to plot ROC curve and the regplot package to construct a nomogram. Time-dependent ROC curves and calibration curves were performed to estimate the predictive accuracy of the nomogram. All data analysis was conducted in R and Rstudio software and p < 0.05 was considered as statistically significance.

**Statistical Analysis**

All experiments were repeated at least 3 times unless otherwise indicated. Data were shown as mean ±SD and were analyzed with GraphPad Prism software or SPSS 26.0. The variance was tested using the Student’s t test, Wilcoxon test, one- or two-way ANOVA depending on the dataset as described in figure legends. Differences were considered significant at p<0.05.

**References:**

1. Lu M, Zhu WW, Wang X, et al. ACOT12-Dependent Alteration of Acetyl-CoA Drives Hepatocellular Carcinoma Metastasis by Epigenetic Induction of Epithelial-Mesenchymal Transition. Cell Metab. 2019;29(4):886-900 e5.

2. Yi C, Chen L, Lin Z, et al. Lenvatinib Targets FGF Receptor 4 to Enhance Antitumor Immune Response of Anti-Programmed Cell Death-1 in HCC. Hepatology. 2021;74(5):2544-60.
